# CircFOXO3 protects against osteoarthritis by targeting its parental gene FOXO3 and activating PI3K/AKT-mediated autophagy

**DOI:** 10.1038/s41419-022-05390-8

**Published:** 2022-11-07

**Authors:** Chen Zhao, Xiaodong Li, Guantong Sun, Pengcheng Liu, Keyu Kong, Xuzhuo Chen, Fei Yang, Xiaoqing Wang

**Affiliations:** 1 Department of Orthopedics, Shanghai Key Laboratory of Orthopedic Implant, Shanghai Ninth People’s Hospital, Shanghai Jiao Tong University School of Medicine, Shanghai, 200011 China; 2 Department of Oral Surgery, Shanghai Key Laboratory of Stomatology & Shanghai Research Institute of Stomatology, National Clinical Research Center for Oral Diseases, Shanghai Ninth People’s Hospital, College of Stomatology, Shanghai Jiao Tong University School of Medicine, Shanghai, 200011 China

**Keywords:** Macroautophagy, Apoptosis

## Abstract

Osteoarthritis (OA) is a degenerative joint disorder causing pain and functional disability. Emerging evidence reveals that circular RNAs (circRNAs) play essential roles in OA progression and development. This study aimed to investigate the role of a novel circRNA factor, circFOXO3, in the progression of OA and elucidate its underlying molecular mechanism. The function of circFOXO3 in OA and interaction between circFOXO3 and its downstream mRNA target, forkhead box O3 (FOXO3), were evaluated by western blot (WB), immunofluorescence (IF), RNA immunoprecipitation, reverse transcription-quantitative PCR (RT-qPCR), and fluorescence in situ hybridization (FISH). Upregulation of circFOXO3 and autophagic flux were detected both in vivo and in vitro by WB, transmission electron microscopy (TEM), IF, and immunohistochemistry (IHC). A mouse model of OA was also used to confirm the role of circFOXO3 in OA pathogenesis in vivo. Decreased expression of circFOXO3 in OA cartilage tissues was directly associated with excessive apoptosis and imbalance between anabolic and catabolic factors of the extracellular matrix (ECM). Mechanistically, circFOXO3 functioned in cartilage by targeting its parental gene FOXO3 and activating autophagy. Intra-articular injection of lentivirus-circFOXO3 alleviated OA in the mouse model. In conclusion, our results reveal the key role played by circFOXO3 in OA progression; circFOXO3 overexpression may alleviate apoptosis of chondrocytes and promote anabolism of the ECM via activation of FOXO3 and autophagy, providing a potentially effective novel therapeutic strategy for OA.

## Introduction

Osteoarthritis (OA) is a degenerative joint disease associated with articular cartilage degeneration, subchondral bone sclerosis, and osteophyte formation, and is the most common joint disease worldwide [1, 2]. According to a World Health Organization survey, approximately 10% of the global population suffers from varying degrees of OA. Moreover, OA, cardiovascular disease, and tumors are ranked as the three major causes of death affecting human health today [3]. Risk factors for OA include advanced age, female sex, previous joint damage, obesity, genetic susceptibility, and congenital deformities [2, 4, 5]. Additionally, mitochondrial dysfunction, oxidative stress, and reduced autophagy can alter chondrocyte function, promoting catabolic processes and cell death during anabolic processes [6, 7]. Therefore, advancing our understanding of the specific molecular mechanisms involved in the initiation and progression of OA is critical for improving prognosis and developing effective treatments.

Recently, several studies have revealed the involvement of non-coding RNAs, including circular RNAs (circRNAs), that are widely present in the human transcriptome, in the occurrence and development of human diseases [8]. CircRNAs are characterized by covalently closed continuous structures with neither 5′ caps nor 3′ polyadenylated tails, unlike linear RNAs. These molecules represent a new class of non-coding RNAs. Due to their unique structure, circRNAs are highly stable, mainly distributed in the cytoplasm, and highly conserved across species [9]. Recently, various circRNAs have been implicated in OA. These circRNAs play a protective role in chondrocytes by participating in multiple pathways in OA, including resisting oxidative stress and inhibiting apoptosis, to protect articular cartilage [10, 11]. However, few circRNAs have been fully studied in articular cartilage. Novel insights into OA treatment are expected through an in-depth exploration of unknown circRNAs in chondrocytes.

Among the many mechanisms that regulate circRNAs, the regulation of autophagy has attracted our attention, because the weakening of autophagic activity is an important contributor to OA development [12, 13]. Autophagy involves the clearance of damaged and dysfunctional macromolecules and organelles. It is a cellular homeostatic mechanism that plays an important role in energy and nutrient regulation [14]. At the cellular level, dysregulation of autophagy leads to oxidative stress disorders, abnormal gene expression, and abnormal cell death [15, 16]. Therefore, identifying molecular targets with important regulatory effects on autophagy may benefit OA treatment by improving autophagy in chondrocytes. CircRNAs are key regulators of autophagy-related regulatory networks in cancer, metabolic diseases, and cerebrovascular diseases [12, 17, 18]. Therefore, we hypothesized that the regulation of autophagy by circRNAs also involved in OA.

CircFOXO3 (has_circ_0006404, mmu_circ_0002207) is a newly discovered circRNA derived from the parent gene forkhead box O3 (FOXO3) and is involved in the regulation of apoptosis and cell cycle. In cardiomyocytes, circFOXO3 binds to the inhibitor of DNA binding 1, E2F transcription factor-1 (E2F1), focal adhesion kinase, and hypoxia-inducible factor-1 alpha in the cytoplasm, affecting their regulation of stress-related pathways and promoting the aging of cardiomyocytes [19]. Among them, the transcription factor E2F1 interacts with FOXO3 and participates in the regulation of the cell cycle and apoptosis [19]. In tumor cells, FOXO3 protein binds to circFOXO3, inhibiting the binding of FOXO3 to E3 ubiquitin-protein ligase MDM2 (MDM2), and promoting the enrichment of FOXO3 protein, thereby inducing tumor cell apoptosis [20]. The progression of OA is often accompanied by the weakening of autophagic activity and resulting increase in the level of apoptosis [6, 13, 21, 22]. FOXO3 is involved in the regulation of autophagy [23, 24]; however, the role of circFOXO3 in chondrocytes has not yet been studied. Furthermore, circFOXO3 is involved in autophagy in various tissues, including the brain [12] and myocardia [19]. Moreover, it is also involved in autophagy regulation in cancer [25, 26]. However, the role of circFOXO3 in the regulation of OA progression through autophagy mechanisms is unknown.

In this study, we quantitatively analyzed the content of circFOXO3 in articular cartilage both in vitro and in vivo using reverse transcription-quantitative polymerase chain reaction (RT-qPCR) and fluorescence in situ hybridization (FISH) experiments, respectively. Subsequently, we elucidated the functional role and underlying molecular mechanisms of circFOXO3 in OA progression. This study paves the way for future research on circRNAs as therapeutic targets for OA and an in-depth understanding of their mechanisms of action.

## Materials and methods

### Reagents

Recombinant mouse interleukin-1 beta (IL-1β) was acquired from Peprotech (Cranbury, NJ, USA), and dissolved in sterile phosphate-buffered saline (PBS) containing 0.1% bovine serum albumin (BSA; Beyotime Biotechnology, Shanghai, China) at a concentration of 10 µg/ml. Primary antibodies against BCL-2 (B-cell lymphoma 2; no. 124; rabbit monoclonal), BAX (BCL-2-associated X; no. D2E11; rabbit monoclonal), Cleaved caspase-3 (no. Asp175; rabbit monoclonal), cleaved poly(ADP-ribose) polymerase (PARP; no. Asp214; rabbit monoclonal), Beclin1 (no. D40C5; rabbit monoclonal), and β-actin (no. D6A8; rabbit monoclonal) were purchased from Cell Signaling Technology (Danvers, MA, USA). Abcam (Cambridge, UK) provided the antibodies against matrix metalloprotease (MMP13; ab39012), a disintegrin and metalloproteinase with thrombospondin motifs 5 (ADAMTS5; ab41037), and collagen II (ab239007). While LC3 antibody (L8918) was purchased from Sigma-Aldrich.

### Animal models

Eight-week-old male C57BL/6 mice (*n* = 48) were used for in vivo experiments. To create a post-traumatic OA model (positive control group), we performed medial meniscus destabilization (DMM) surgery as previously described [27, 28]. Mice were randomly divided into four groups with six mice per group: sham + vector, DMM + vector, DMM + control lentivirus (Lv-NC), and DMM + circFOXO3 lentivirus (Lv-circFOXO3). Briefly, mice were anesthetized, and their medial joint capsules were incised to expose the medial meniscotibial ligament (MMTL). The MMTL was then severed using microsurgical scissors to release the ligaments attached to the tibial plateau, thereby destabilizing the medial meniscus. After surgery, the incision was closed using sutures and disinfected. A sham operation was performed in control animals in parallel by incising the medial knee joint capsule. Two days following the surgery, 10 µl of lentivirus was injected into the articular cavity via the trans-patella tendon approach. Eight weeks after the operation, the mice were sacrificed under anesthesia, and their knee joints were dissected and processed for imaging and histological evaluation.

Afterwards, to investigate whether the effect of circFOXO3 on osteoarthritis progression and cartilage degeneration is FOXO3-dependent, the recombinant adenovirus shFOXO3 was constructed and packaged by OBiO Technology (Shanghai, China). The mice were randomly divided into four groups (sham group, DMM group, DMM + circFOXO3 group and combined circFOXO3 overexpression and FOXO3 knockdown group) with six mice in each group. Two days after the DMM surgery, the stock solutions containing above virus (approximately 1 × 10^10^ plaque formation unit (PFU)/ml) were slowly injected into right knee joint cavities without solution spill. The subsequent process was the same as above.

### ATDC5 cell culture

The ATDC5 cell line was used as a cellular model to assess chondrogenesis in vitro [29]. The chondrogenic cell line ATDC5 was cultured as previously described [30]. Mouse ATDC5 immortalized chondrocytes were purchased from Chinese Academy of Sciences (Shang’hai, China) and were maintained in DMEM/F12 supplemented with 5% fetal bovine serum (FBS) and 1% penicillin-streptomycin (Gibco; Thermo Fisher Scientific, Waltham, MA, USA) at 37 °C and 5% CO_2_. Before further experimental processing, ATDC5 cells were treated with insulin-transferrin-selenium (ITS) (Gibco) for 14 days.

### ATDC5 cell treatment

To explore the expression level of circFOXO3-FOXO3 in ATDC5 chondrocytes, the chondrocytes were exposed to varying concentrations of IL-1β (0, 5, 10, and 20 ng/ml) for 24 h and 10 µM of IL-1β for different durations (0, 24, 48, and 72 h). In the functional study of circFOXO3 in vitro, the chondrocytes were pre-exposed to Lv-circFOXO3 or small-interfering RNA (siRNA) targeting circFOXO3 (si-circFOXO3) followed by treatment with IL-1β (10 ng/ml) for 24 h. To assess the involvement of autophagy in circFOXO3-mediated OA, ATDC5 chondrocytes were pretreated with Lv-circFOXO3 and IL-1β (10 ng/ml) for 24 h.

### Lentivirus transfection

The Lv-circFOXO3, Lv-FOXO3, and Lv-NC lentiviruses were purchased from OBiO Technology (Shanghai, China). Cells were transfected at 30–50% confluency. After 12 h, >95% of cells were viable. The medium was changed, and cells were passaged after three days. After storing a portion of the cells, the remaining cells were used for further experiments. Transfection efficiency was assessed by western blotting.

### siRNA transfection

The si-circFOXO3, si-FOXO3, and si-NC siRNAs were acquired from Genomeditech (Shanghai, China). Cells were inoculated and cultured in six-well plates for 24 h to achieve a cell density of 60–70%. Next, 50 nM of control or siRNA duplexes were added according to the Lipofectamine 3000 siRNA transfection system (Thermo Fisher Scientific, Waltham, MA, USA). The gene knockdown effect was verified by western blot experiments.

### Western blot analysis

Cultured cells were lysed using RIPA lysis buffer supplemented with phosphatase and protease inhibitors (Roche Diagnostics, Basel, Switzerland). Total protein was quantified using a bicinchoninic acid assay (Thermo Fisher Scientific) and then equal quantities of extracted protein (20–30 µg) were separated via 12 or 15% sodium dodecyl sulfate-polyacrylamide gel electrophoresis and electroblotted onto 0.22-µm PVDF membranes (MilliporeSigma, Burlington, MA, USA). The membranes were blocked using 5% BSA-PBS (Beyotime Biotechnology) at room temperature (RT = 25 °C) for 1 h, and then incubated with primary antibodies (Cleaved caspase-3, Cleaved PARP, BAX, BCL-2, Collagen II, MMP13, ADAMTS5). The membranes were washed with Tris-buffered saline (TBS)-0.1% Tween 20 (TBST) and subsequently incubated with anti-rabbit IgG (H + L) secondary antibody (cat. no. 5151; DyLight™ 800 4X PEG Conjugate; Cell Signaling Technology; 1:5000) for 1 h at RT in the dark. After washing in TBST, protein immunoreactivity was detected using the Odyssey Fluorescence Imaging system (LI-COR Biosciences, Lincoln, NE, USA). Semi-quantitative analysis of protein band intensity was conducted using the ImageJ v1.8.0 software (National Institutes of Health) and normalized to the intensity of the internal loading control, β-actin.

### RNA extraction and RT-qPCR

Total RNA was extracted from cultured cells using TRIzol® reagent (Invitrogen, Waltham, MA, USA) according to the manufacturer’s instructions. Complementary DNA (cDNA) was reverse transcribed using TaKaRa reverse transcription reagents (TaKaRa Bio Inc., Kusatsu, Shiga, Japan) and RT-qPCR was performed using the Real-Time PCR Mix (TaKaRa Bio Inc.) on a light cycler (Roche Diagnostics) with the following primers: circFOXO3 (forward primer 5′-GTGGGGAACTTCACTGGTGCTAAG-3′ and reverse primer 5′-GGGTTGATGATCCACCAAGAGCTCTT-3′), FOXO3 (forward primer 5′-AAACGGCTCACTTTGTCCCAGATC-3′ and reverse primer 5′-CCTCGGCTCTTGGTGTACTTGTTG-3′), autophagy-related gene 5 (ATG5; forward primer 5′-CATCCACTGGAAGAATGACAG-3′ and reverse primer 5′-TGATGCAAGAAGATCAAATAG-3′), Beclin1 (forward primer 5′-TTTTCTGGACTGTGTGCAGC-3′ and reverse primer 5′-GCTTTTGTCCACTGCTCCTC-3′), microtubule-associated proteins 1A/1B light chain (LC3; forward primer 5′-GATGTCCGACTTATTCGAGAGC-3′ and reverse primer 5′-TTGAGCTGTAAGCGCCTTCTA-3′), and glyceraldehyde-3-phosphate dehydrogenase (GAPDH; forward primer 5′-CGACTTCAACAGCAACTCCCACTCTTCC-3′ and reverse primer 5′-TGGGTGGTCCAGGGTTTCTTACTCCTT-3′). Target gene expression levels were determined using the 2^-ΔΔCq^ method [31], with GAPDH as the internal reference control.

### RNA treatment with RNase R and PCR

For samples requiring linear RNA depletion, total RNA (2 mg) was treated with or without 3 U/mg RNase R (RNR07250; Epicentre Technologies-Illumina, San Diego, CA, USA) at 37 °C for 20 min. RT-qPCR was then performed. The relative expression of circFoxO3 or FOXO3 was quantified using the comparative CT method (2^-ΔΔCq^) and normalized to GAPDH.

### High-density culture

To assess chondrogenic differentiation, 1.5 × 10^5^ ATDC5 were resuspended in 10 µl incomplete MEM/F12 (Gibco) and seeded as micromasses at the bottom of a 24-well plate. The cells were allowed to adhere for 1 h at 37 °C, after which 0.5 ml MEM/F12 containing 10 ng/ml ITS and 2% FBS were added. All media were refreshed on alternate days, and after 14 days, the micromasses were stained with Alcian blue for 24 h at RT. Digital images were captured using a light microscope at ×7.8 magnification (DM4000 B; Leica Microsystems GmbH, Wetzlar, Germany).

### Terminal deoxynucleotidyl transferase deoxyuridine triphosphate nick-end labeling (TUNEL) staining assay

TUNEL staining was performed using the colorimetric TUNEL apoptosis assay kit (Beyotime Biotechnology) according to the manufacturer’s instructions. Briefly, cartilage specimens or cells were fixed in 4% paraformaldehyde, embedded in paraffin, and sectioned at 5 μm. The sections were then deparaffinized in xylene and ethanol and rehydrated with proteinase K. After washing thrice with PBS, the sections were incubated with the TUNEL reaction mixture for 2 h at 37 °C in a moist chamber. The nuclei were stained using DAPI. All images were acquired using a DM4000 B epifluorescence microscope (Leica Microsystems GmbH).

### Cell counting kit-8 (CCK-8) cell proliferation assay

For the cell proliferation assay, cells were transfected with siRNA or lentivirus for 24 h, then seeded onto 96-well plates at a density of 1 × 10^4^ cells/well and incubated for 24 h. At the end time point, cell proliferation was detected using a CCK-8 assay (Sigma-Aldrich, St. Louis, MO, USA). Absorbance at 450 nm (mean optical density) was measured using an Infinite M200 Pro multimode microplate reader (Tecan Group, Ltd., Männedorf, Switzerland).

### Transmission electron microscopy (TEM)

Autophagic vacuoles in ATDC5 cells were imaged using TEM. In brief, the samples were fixed with 2.5% glutaraldehyde and 1% osmium tetroxide in 0.2 mol/l sodium phosphate buffer for 12 h. After dehydration in graded ethanol (50, 75, and 95%), the samples were cut into 70-nm-thick sections and stained using uranyl acetate and lead citrate. The autophagic vacuoles were imaged using a JEM-1400 electron microscope (JEOL Ltd., Akishima, Tokyo, Japan).

### FISH

Cy-3-labeled circFOXO3 probes were constructed by Servicebio. (Wuhan, China). Cells were seeded in 12-well plates with sterile glass lids and processed overnight. Cells were then fixed with PBS containing 37% formaldehyde for 15 min at 25 °C and dehydrated with 70% ethanol for 1 h at 4 °C. Slides were hybridized for 14–16 h at 37 °C. Hybridization buffer for RNA-FISH dissolves the probe at a concentration of 20 nM. After 6–8 h hybridization, the slides were washed with 10% formamide/2X saline-sodium citrate for 30 min on a shaker at 37 °C, and washed thrice with PBS and 0.1% (v/v) Tween 20. Cells were then stained using Alexa Fluor 546-conjugated streptavidin for 1 h at RT. Coverslips were re-stained with DAPI, mounted with ProLong Gold antifade reagent, and imaged using a DM4000 B epifluorescence microscope (Leica Microsystems GmbH). For FISH of in vivo samples, biopsies were performed at 37 °C under 0.8% pepsin treatment prior to hybridization. Sections were then dewaxed, rehydrated, and infiltrated for 30 min.

### Immunofluorescence (IF)

For IF assessment, ATDC5 cells were cultured on slides seeded on a six-well plate. At 10% confluence, the cells were stimulated with IL-1β for 24 min at 37 °C, with or without lentivirus or siRNA pretreatment. The slides were then fixed using 4% paraformaldehyde at RT for 48 h, and then immersed in PBS (pH 7.4) and washed thrice for 5 min each. Autofluorescence quencher was added to the sections for 5 min, which were then blocked using a blocking buffer (Cell Signaling Technology) for 30 min at RT. The slides were subsequently incubated with primary antibodies in a wet box at 4 °C overnight. Anti-collagen II, anti-MMP13, or anti-LC3B primary antibody was used at a 1:100 dilution. The following day, the slides were washed using PBS and incubated with a recombinant Alexa Fluor 555 anti-mannose-6-phosphate receptor antibody (ab203438) for 50 min at RT in the dark. Subsequently, the slides were washed using PBS and incubated with DAPI solution (Merck KGaA, Darmstadt, Germany) for 5 min at RT in the dark to stain cell nuclei. After a final PBS wash, the samples were air-dried and sealed with anti-fluorescence quenching tablets. Digital fluorescence images were captured using a DM4000 B epifluorescence microscope (Leica Microsystems GmbH) at ×10 and ×20 magnifications, and interocular distance measurements were obtained using the Image Pro Plus 6.0 software (Media Cybernetics, Inc., Rockville, MD, USA).

### RNA immunoprecipitation (RIP)

RIP experiments were performed using the Magna RIP RNA-Binding Protein Immunoprecipitation Kit (MilliporeSigma). Approximately 1 × 10^7^ cells were sedimented and resuspended with an equal pellet volume of RIPA lysis buffer supplemented with a protease inhibitor cocktail and RNase inhibitors. Cell lysates were incubated with 5 µg of antibody against FOXO3 (no. 75D8; rabbit monoclonal) or control IgG-coated beads and mixed by rotation at 4 °C overnight. After treating with proteinase K buffer, the immunoprecipitated RNAs were extracted using a RNeasy MinElute Cleanup Kit (Qiagen, Hilden, Germany) and reverse transcribed using the Prime-Script RT Master Mix (TaKaRa Bio Inc.). The abundance of circFOXO3 was detected using RT-qPCR.

### Radiographic analysis

Digital X-ray imaging of the right lower limbs was performed according to the manufacturer’s instructions [32] in the anteroposterior axis with a 21 lp/mm detector that provided up to 5X geometric magnification (Faxitron VersaVision; Faxitron Bioptics LLC, Tucson, AZ, USA).

### Micro-computed tomography (micro-CT) scanning

A high-resolution micro-CT scanner (μCT-100, SCANCO Medical AG, Wangen-Brüttisellen, Switzerland) was used to perform the micro-CT scanning. The μCT-100 software (v6.5-3, SCANCO Medical AG) was used for three-dimensional (3D) knee reconstruction and image capture.

### Histological staining

Fixed lower limb samples were embedded in paraffin and histologically sectioned (5-µm thickness). For histological assessment, paraffin-embedded tissue sections were processed for safranin-O-fast green and hematoxylin and eosin (H&E) staining (Servicebio Technology Co., Wuhan, Hubei, China) at RT for 2–5 min, according to the manufacturer’s instructions. The Osteoarthritis Research Society International (OARSI) score was based on the safranin-O-fast green staining of each specimen [33]: 0, normal; 0.5, loss of safranin-O without structural changes; 1, small fibrillations without loss of cartilage; 2, vertical clefts down to the layer immediately below the superficial layer and some loss of surface lamina; 3, vertical clefts/erosion to the calcified cartilage extending to <25% of the articular surface; 4, vertical clefts/erosion to the calcified cartilage extending to 25–50% of the articular surface; 5, vertical clefts/erosion to the calcified cartilage extending to 50–75% of the articular surface; and 6, vertical clefts/erosion to the calcified cartilage extending to >75% of the articular surface.

### Immunohistochemistry

Fixed lower limb samples were embedded in paraffin as described above, sectioned into 8-µm slices, and subjected to an immunohistochemistry kit (cat. no. G1215-200T; Servicebio Technology Co.) according to the manufacturer’s instructions. The sections were incubated with primary antibodies (COL2, MMP13 and LC3) at 4 °C overnight. The following day, the slides were washed using PBS and incubated with goat anti-mouse/rabbit IgG horseradish peroxidase-polymer (cat. no. 91196; anti-rabbit; 1:500; Cell Signaling Technology) for 30 min at RT using 3,3′-diaminobenzidine as the chromogen. Digital images were captured using a DM4000 B microscope (Leica Microsystems GmbH) at ×10 and ×20 magnification, and positively stained cell measurements were obtained using the Image Pro Plus 6.0 software.

### Statistical analysis

All data were collected from at least three independent experiments or repeated measures. Differences between two groups were analyzed using the Student’s *t*-test while those between multiple groups were analyzed using one-way analysis of variance and Tukey’s multiple comparison test. The results are presented as the mean ± standard deviation, and statistical significance was set at *p* < 0.05. All statistical analyses were performed using the Graph Pad Prism 9.0 software.

## Results

### CircFOXO3 expression is lower in OA tissue than in control and is predominantly localized in the cytoplasm

**Fig. 1:**
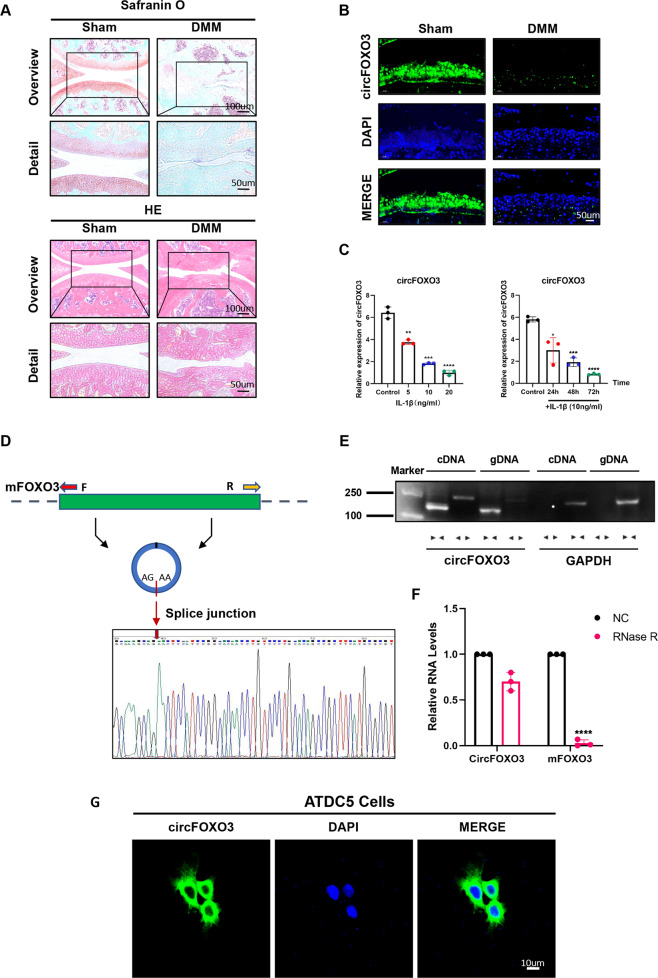
CircFOXO3 exhibits lower expression in OA tissue and predominantly localized in the cytoplasm. **A** Safranin-O/fast green staining and H&E staining of the cartilage from different groups. Scale bars, 100 µm and 50 µm. **B** Expression levels of circFOXO3 was detected by RNA FISH in C57BL/6 samples. Representative photomicrographs and fluorescence intensity of FISH are shown. Scale bar, 50 µm. **C** Expression of circFOXO3 after treating with IL-1β for different concentrations and time spans. (*n* = 3) *p < 0.05, **p < 0.01, ***p < 0.001, ****p < 0.0001. **D** Sanger sequencing after PCR using the indicated divergent flanking primers confirmed the “head-to-tail” splicing of circFOXO3. **E** The presence of circFOXO3 was validated in ATDC5s, circFOXO3 was amplified by divergent primers in cDNA but not gDNA. GAPDH was used as a negative control. **F** After treating or without treating with RNase R, the expression of circFOXO3 and FOXO3 mRNA in ATDC5s were detected by RT-qPCR. (*n* = 3) ****p < 0.0001. **G** RNA FISH showed that circFOXO3 was predominantly localized in the cytoplasm. CircFOXO3 probes were labeled with Cy-3. The nuclei were stained with DAPI. Scale bar, 10 µm.

We detected the expression of circFOXO3 in a mouse model of OA cartilage caused by destabilization via DMM surgery. Eight weeks after surgery, we observed decreased proteoglycan staining (red) and a rough surface in the articular cartilage in OA mice compared with that in sham mice, suggesting cartilage matrix degeneration (Fig. 1A). Moreover, RNA-FISH of cartilage sections indicated that the increased degradation of cartilage corresponded to decreased expression of circFOXO3 in chondrocytes in OA animals compared with that in the sham group (Fig. 1B). Considering the correlation between joint damage and the degree of arthritis and inflammation, we hypothesized that the expression level of circFOXO3 is also reduced in an inflammatory environment. This hypothesis was confirmed by RT-qPCR analysis which detected downregulated circFOXO3 RNA levels in the ATDC5 chondrocytes in vitro, whether treated with different concentrations of IL-1β for 24 h or with 10 ng/ml IL-1β for different time periods, compared with that in the control (Fig. 1C). Taken together, these results revealed that circFOXO3 expression was negatively associated with OA severity.

Comparison between the circFOXO3 sequences acquired from circBase (mmu_circ_0002207) and FOXO3 mRNA sequences revealed that circFOXO3 was looped and comprised exon 2 of its parental gene, and its head-to-tail splicing was further confirmed by Sanger sequencing (Fig. 1D). However, head-to-tail splicing may be produced by trans-splicing or genomic rearrangement. For the next experiments, it was necessary to rule out these two possibilities. Therefore, we further designed convergent primers to amplify FOXO3 mRNA and divergent primers to amplify circFOXO3. CircFOXO3 was shown to only be amplified by divergent primers and was detected in cDNA but not in genomic DNA (gDNA), whereas FOXO3 mRNA was amplified by convergent primers both in cDNA and gDNA (Fig. 1E). Furthermore, the mRNA level of FOXO3 was significantly downregulated after RNase R treatment compared with that in the control, but circFOXO3 was resistant to RNase R treatment due to its covalently closed-loop structure (Fig. 1F). RNA-FISH experiments revealed that circFOXO3 was predominantly localized in the cytoplasm rather than the nucleus (Fig. 1G). Together, these results indicate that the expression of the circFOXO3 is downregulated with the progression of OA and inflammation, and is primarily localized in the cytoplasm, thus circFOXO3 may play a role in regulating OA progression.

### CircFOXO3 regulates cell viability, apoptosis, and extracellular matrix (ECM) metabolism in ATDC5 chondrocytes

**Fig. 2:**
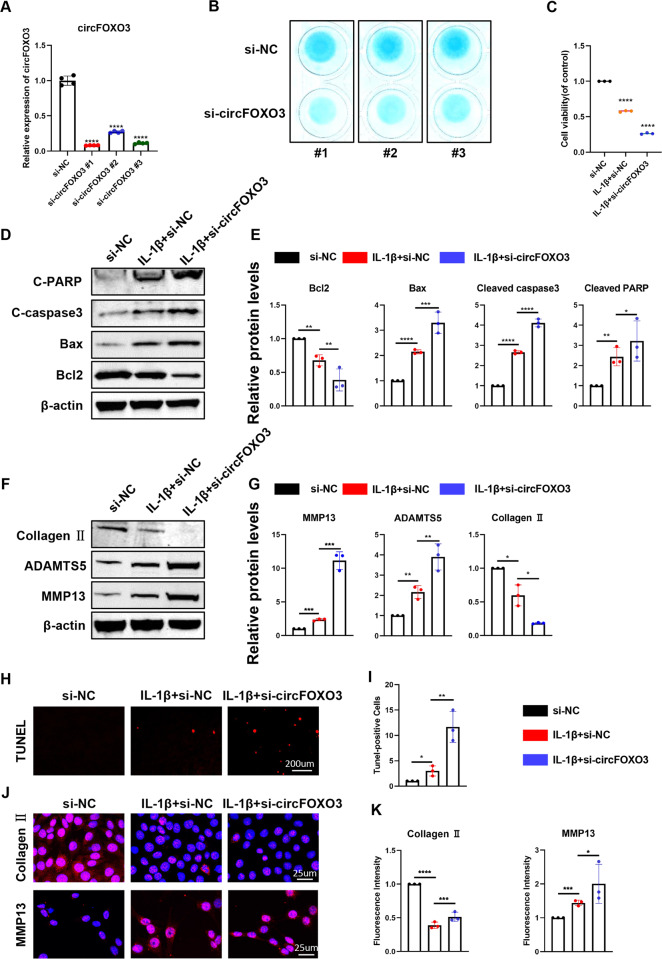
Knockdown of circFOXO3 on cell viability, apoptosis and ECM metabolism in ATDC5 chondrocytes. **A** The knockdown efficiency of circFOXO3 in ATDC5 chondrocytes was detected by RT-qPCR. (*n* = 4) ****p < 0.0001. **B** Alcian blue staining of siRNA-treated ATDC5 chondrocytes. **C** Cell viability determined by CCK-8 assay. (*n* = 3) ****p < 0.0001. **D** Western blot analysis of Cleaved PARP, Cleaved caspase-3, Bax, and Bcl2 when circFOXO3 was downregulated in ATDC5 chondrocytes. **E** The optical density analysis was performed from the results of three independent experiments of western blot samples. *p < 0.05, **p < 0.01, ***p < 0.001, ****p < 0.0001. **F** Western blot analysis of MMP13, ADAMTS5 and Collagen II when circFOXO3 was downregulated in ATDC5 chondrocytes. **G** The optical density analysis was performed from the results of three independent experiments of western blot samples. *p < 0.05, **p < 0.01, ***p < 0.001. **H**, **I** ATDC5s proliferation activity was detected by TUNEL staining when circFOXO3 was downregulated. Representative photomicrographs and quantitative data showing the percentage of TUNEL-positive cells are shown. (n = 3) Scale bar, 200 µm. *p < 0.05, **p < 0.01. **J**, **K** Representative photomicrographs and fluorescence intensity of IF of MMP13, and Collagen II in ATDC5s after treating with IL-1β (10 ng/ml) for 24 h and the worsening effects of knockdown circFOXO3 on IL-1β. (*n* = 3) Scale bar, 25 µm. *p < 0.05, ***p < 0.001, ****p < 0.0001.

To investigate the role of circFOXO3 in regulating matrix-degrading enzymes and apoptosis, we transfected ATDC5 chondrocytes with three circFOXO3 siRNAs. RT-qPCR confirmed the success of circFOXO3 knockdown (Fig. 2A).

The inhibition of circFOXO3 revealed disturbances in ATDC5 chondrocyte function and reduced proteoglycan synthesis assessed by high-density cell culture and Alcian blue staining compared with that in controls (Fig. 2B). We then assessed the effect of circFOXO3 on ATDC5 chondrocyte viability using the CCK-8 assay. The results showed that knockdown of circFOXO3 expression reduced chondrocyte viability compared with that in the control (Fig. 2C). OA is a well-known disease closely related to apoptosis. Therefore, the effects of circFOXO3 inhibition on IL-1β-induced ATDC5 apoptosis were further examined. As shown in Fig. 2D, E, inhibition of circFOXO3 significantly enhanced the expression of Cleaved PARP, Cleaved caspase-3, and BAX compared with that in controls, whereas the expression of BCL-2 was downregulated in knockdown ATDC5 chondrocytes. TUNEL staining further demonstrated that circFOXO3 regulates apoptosis (Fig. 2H, I).

We also found that circFOXO3 deficiency significantly downregulated Collagen II expression, whereas protein levels of the matrix catabolic enzymes MMP13 and ADAMTS5 were significantly upregulated in knockdown ATDC5 chondrocytes compared with those in the controls (Fig. 2F, G). IF further demonstrated that circFOXO3 knockdown affected IL-1β-stimulated collagen II and MMP13 expression in ATDC5 cells (Fig. 2J, K). These data suggest that circFOXO3 knockdown promotes cell apoptosis and catabolism in ATDC5 chondrocytes.

**Fig. 3:**
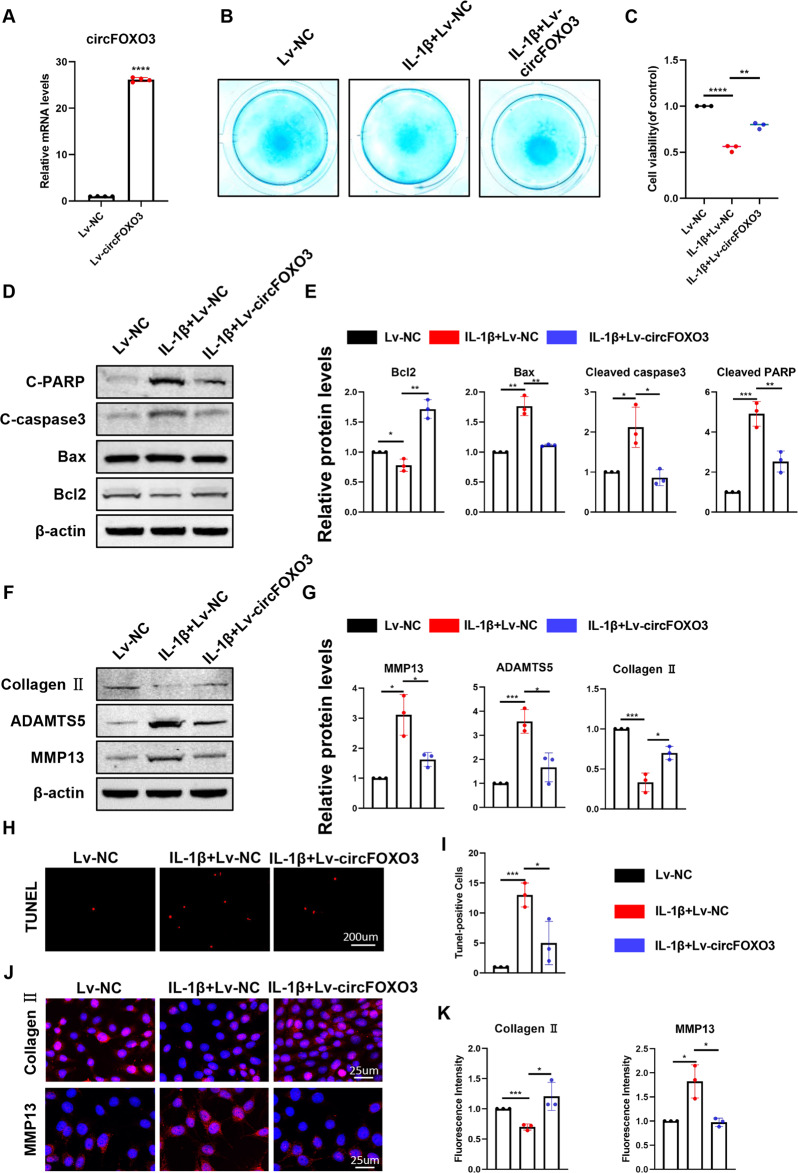
Overexpression of circFOXO3 on cell viability, apoptosis and ECM metabolism in ATDC5 chondrocytes. **A** The overexpression efficiency of circFOXO3 in ATDC5s detected by qRT-PCR. (*n* = 4) ****p < 0.0001. **B** Alcian blue staining of lentivirus-treated ATDC5 chondrocytes. **C** Cell viability determined by CCK-8 assay. (n = 3) **p < 0.01, ****p < 0.0001. **D**, **E** Western blot analysis of Cleaved PARP, Cleaved caspase-3, Bax and Bcl2 when circFOXO3 was upregulated in ATDC5 chondrocytes. The optical density analysis was performed from the results of three independent experiments of western blot samples. *p < 0.05, **p < 0.01, ***p < 0.001. **F**, **G** Western blot analysis of MMP13, ADAMTS5 and Collagen II when circFOXO3 was upregulated in ATDC5 chondrocytes. The optical density analysis was performed from the results of three independent experiments of western blot samples. *p < 0.05, ***p < 0.001. **H**, **I** ATDC5s proliferation activity was detected by TUNEL staining when circFOXO3 was overexpressed. Representative photomicrographs and quantitative data showing the percentage of TUNEL-positive cells are shown. (*n* = 3) Scale bar, 200 µm. *p < 0.05, ***p < 0.001. **J**, **K** Representative photomicrographs and fluorescence intensity of IF of MMP13, and Collagen II in ATDC5s after treating with IL-1β (10 ng/ml) for 24 h and the saving effects of overexpressed circFOXO3 on IL-1β. (*n* = 3) Scale bar, 25 µm. *p < 0.05, ***p < 0.001.

To further explore the therapeutic effect of circFOXO3, an overexpression virus (lentivirus) was constructed and transfected in ATDC5 chondrocytes to upregulate circFOXO3 expression (Fig. 3A). Decreased proteoglycan synthesis induced by IL-1β stimulation was rescued by overexpression of circFOXO3 (Fig. 3B). We then performed gain-of-function experiments and found that overexpression of circFOXO3 increased the viability of ATDC5 chondrocytes compared with that in the controls, as revealed by the CCK-8 assay (Fig. 3C). Western blot and IF results showed that treatment with IL-1β promoted the expression of BAX, Cleaved caspase-3, Cleaved PARP, MMP13, and ADAMTS5 while decreasing that of BCL-2 and Collagen II compared with that in the controls. Additionally, our results demonstrate that the overexpression of circFOXO3 antagonized the effects of IL-1β (Fig. 3D–G, J, K). TUNEL staining also revealed a rescue effect of circFOXO3 overexpression on apoptosis (Fig. 3H, I). Taken together, these results indicate that circFOXO3 protects against OA by regulating cell proliferation, apoptosis, and ECM metabolism in chondrocytes.

### The downstream molecule FOXO3 regulates apoptosis and ECM metabolism in ATDC5 chondrocytes

To further explore the role of the downstream targeting molecule FOXO3 in OA, we generated three FOXO3 siRNAs and one overexpression virus that specifically knocks down or overexpresses FOXO3 in ATDC5 chondrocytes. Both RT-qPCR and western blotting showed that FOXO3 was successfully knocked down or overexpressed at the RNA and protein levels compared with that in controls (Fig. S1A–C). Using the CCK-8 kit, we found that knockdown or overexpression of FOXO3 also significantly affected cell viability (Fig. S1D). Moreover, proteoglycan synthesis was sharply reduced in si-FOXO3-treated ATDC5 chondrocytes compared with that in controls (Fig. S1E). Our results also showed that FOXO3 deficiency directly promoted apoptosis. As FOXO3 knockdown upregulated the expression levels of apoptosis-associated proteins (BAX, Cleaved caspase-3, and Cleaved PARP) and downregulated the expression of BCL-2, overexpression of FOXO3 significantly reversed the effects of IL-1β simulation (Fig. S1F, G). Thus, the knockdown or overexpression of FOXO3 plays a role in maintaining chondrocyte homeostasis similar to its role in apoptosis (Fig. S1H, I). However, FOXO3 overexpression alone did not alter the expression levels of MMP13, ADAMTS5, or collagen II (Fig. S2). Furthermore, proteoglycan loss induced by IL-1β stimulation was also rescued by overexpression of FOXO3 (Fig. S1J). TUNEL staining confirmed that FOXO3 regulates apoptosis (Figs. S1K, L and S3A, B). IF analysis of collagen II and MMP13 further indicated that FOXO3 mediated the degradation of the ECM (Figs. S1M, N and S3C, D).

### Regulation of OA by circFOXO3 is FOXO3-dependent

**Fig. 4:**
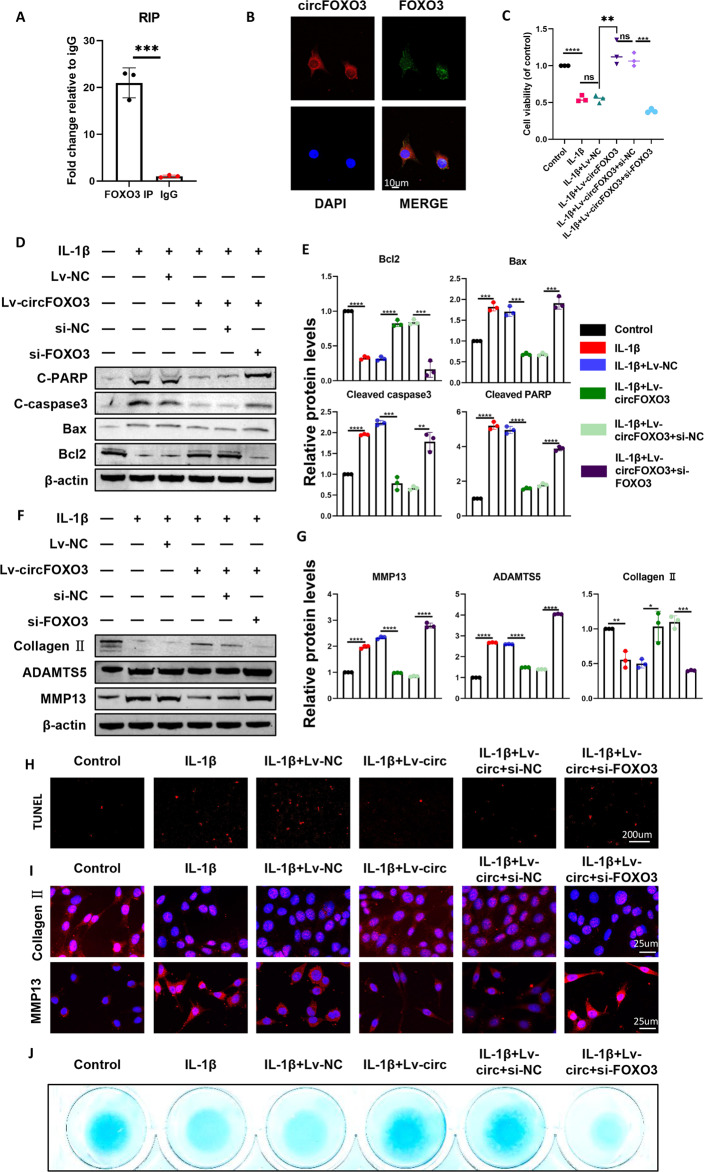
The regulation of circFOXO3 on osteoarthritis is FOXO3-dependent. **A** RIP assay was performed to detect circFOXO3 levels in ATDC5 cells stably expressing FOXO3. (*n* = 3) ***p < 0.001. **B** Representative images of circFOXO3 (red) and FOXO3 (green) labeled fluorescence in situ hybridization (FISH) staining. Scale bar, 10 µm. **C** Cell viability determined by CCK-8 assay. (*n* = 3) **p < 0.01, ***p < 0.001, ****p < 0.0001. **D**, **E** Western blot analysis of Cleaved PARP, Cleaved caspase-3, Bax and Bcl2 when Lv-circFOXO3 and si-FOXO3 were co-transfected. The optical density analysis was performed from the results of three independent experiments of western blot samples. ***p < 0.001, ****p < 0.0001. **F**, **G** Western blot analysis of MMP13, ADAMTS5 and Collagen II when Lv-circFOXO3 and si-FOXO3 were co-transfected. The optical density analysis was performed from the results of three independent experiments of western blot samples. *p < 0.05, **p < 0.01, ***p < 0.001, ****p < 0.0001. **H** TUNEL staining assay was conducted on the ATDC5 chondrocytes, as treated above. Scale bar, 200 µm. **I** Immunofluorescence staining of MMP13 and collagen II in ATDC5 chondrocytes as treated above. Scale bar, 25 µm. **J** Alcian blue staining of Lv-circFOXO3 and si-FOXO3 co-transfected ATDC5 chondrocytes.

As the circFOXO3 level is abundant and stable in the cytoplasm, we further investigated whether the regulation of cellular metabolism by circFOXO3 is achieved by other genes, such as the parental gene of circFOXO3. As predicted, RIP results indicated that circFOXO3 was pulled down by the anti-FOXO3 antibody (Fig. 4A). RNA-protein colocalization in ATDC5 chondrocytes further verified the interaction between FOXO3 and circFOXO3 (Fig. 4B).

To further examine whether circFOXO3 functions in OA via interaction with FOXO3, we co-infected cells with Lv-circFOXO3 and si-FOXO3. Overexpression of circFOXO3 rescued the reduction in cell viability caused by IL-1β, however, the rescue effect of this overexpression on cell viability was reversed by simultaneous FOXO3 knockdown (Fig. 4C). The protein levels of BAX, Cleaved caspase-3, and Cleaved PARP significantly increased while BCL-2 levels decreased in the cells co-infected with Lv-circFOXO3 and si-FOXO3 compared with that in the cells infected with Lv-circFOXO3 alone (Fig. 4D, E). Regarding ECM metabolism, protein levels of MMP13 and ADAMTS5 significantly increased while Collagen II levels decreased in the cells co-infected with Lv-circFOXO3 and si-FOXO3 compared with those in the cells infected with Lv-circFOXO3 alone (Fig. 4F, G). The TUNEL staining results showed that the rescue effect of circFOXO3 overexpression on apoptosis was reversed by FOXO3 knockdown (Figs. 4H and S4A). IF analysis also showed that the expression of COL2A1 decreased and MMP13 levels increased in the cells treated with circFOXO3 and FOXO3 expression inhibitors compared with those in the cells treated with Lv-circFOXO3 (Figs. 4I and S4B, C). The conclusions drawn from high-density cell culture and analysis of proteoglycan synthesis are consistent with the above results (Fig. 4J). Overall, the above results suggest that circFOXO3 regulates apoptosis and extracellular matrix metabolism by targeting FOXO3.

### CircFOXO3 enhances autophagy in IL-1β-treated ATDC5 chondrocytes

**Fig. 5:**
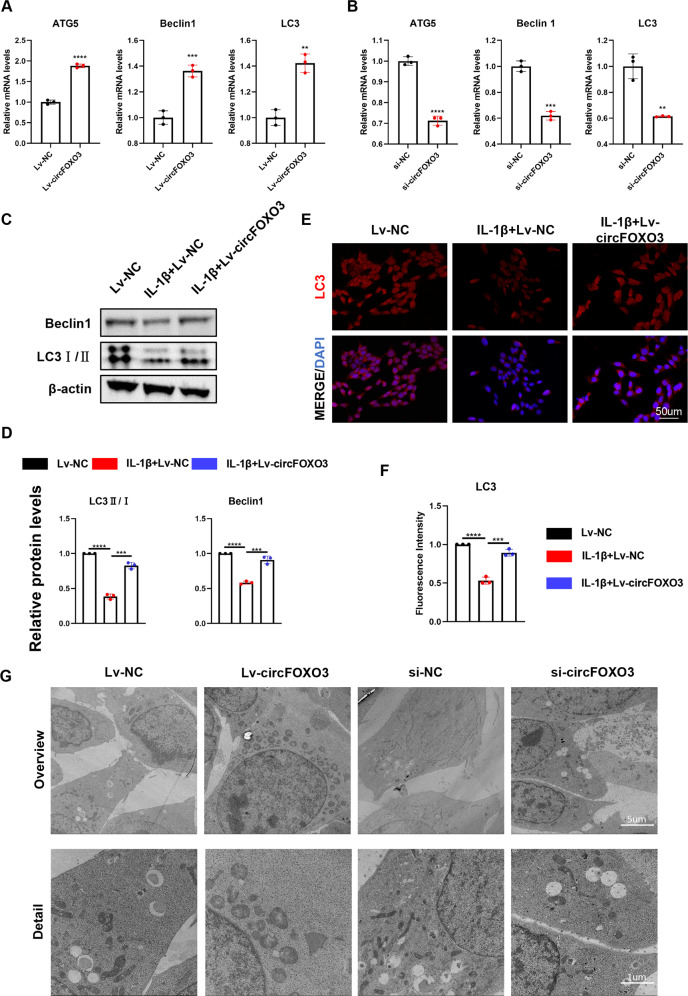
CircFOXO3 enhances autophagy in IL-1β-treated ATDC5 chondrocytes. **A** The expression levels of ATG5, Beclin1 and LC3 was detected by qRT-PCR when circFOXO3 was overexpressed in ATDC5s. (*n* = 3) **p < 0.01, ***p < 0.001, ****p < 0.0001. **B** The expression levels of ATG5, Beclin1 and LC3 was detected by qRT-PCR when circFOXO3 was downregulated in ATDC5s. (*n* = 3) **p < 0.01, ***p < 0.001, ****p < 0.0001. **C, D** Western blot analysis of LC3 and Beclin1 when circFOXO3 was overexpressed in ATDC5 chondrocytes. The optical density analysis was performed from the results of three independent experiments of western blot samples. ***p < 0.001, ****p < 0.0001. **E, F** Representative photomicrographs and fluorescence intensity of IF of LC3 in ATDC5s after treating with IL-1β (10 ng/ml) for 24 h and the saving effects of overexpressed circFOXO3 on IL-1β. (*n* = 3) Scale bar, 50 µm. ***p < 0.001, ****p < 0.0001. **G** Representative TEM images of chondrocytes treated with Lv-circFOXO3 or si-circFOXO3. Scale bars, 5 µm and 1 µm.

Next, we investigated whether the role of circFOXO3 in OA was achieved through the regulation of autophagy. Using RT-qPCR, we found that Lv-circFOXO3-treated ATDC5 cells overexpressed autophagy markers including ATG5, Beclin1, and LC3 (Fig. 5A) compared with that in controls, and overexpressed LC3II and Beclin1 as shown by western blotting (Fig. 5C, D) and IF staining of LC3 (Fig. 5E, F). TEM confirmed that Lv-circFOXO3 increased the number of autophagy-associated vesicles compared with that in the control (Fig. 5G). In contrast, knockdown of circFOXO3 not only downregulated the expression levels of autophagy markers (Fig. 5B), but also reduced the formation of autophagy-related vesicles at the electron microscopy level compared with that in the control (Fig. 5G). These results suggest that the anti-apoptotic activity and function of circFOXO3 in regulating ECM metabolism may be related to the promotion of autophagy.

### CircFOXO3 autophagy sensitively regulates the IL-1β-induced ATDC5 chondrocyte arthritis phenotype

**Fig. 6:**
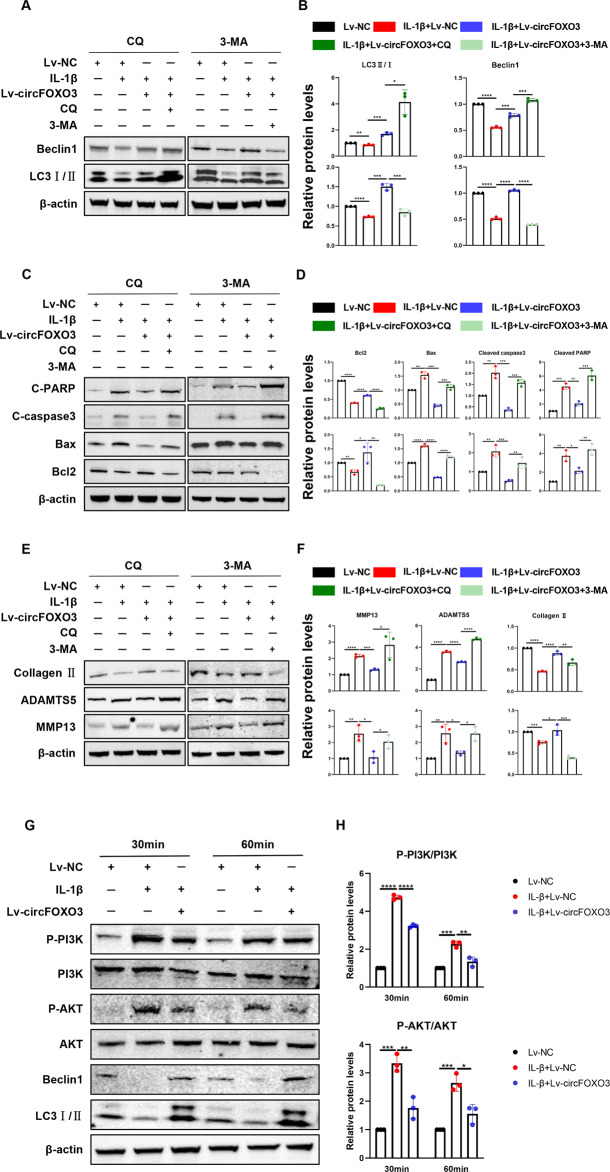
CircFOXO3 autophagy sensitively regulates IL-1β-induced ATDC5 chondrocyte arthritis phenotype. **A** Representative Western blot of Beclin1 and LC3 in ATDC5 chondrocytes pretreated with Lv-circFOXO3 with or without IL-1β (10 ng/mL) in the presence or absence of CQ (50 μM) or 3-MA (5 mM) for 24 h. **B** quantitative analysis of the protein levels in Fig. 7B. (n = 3) *p < 0.05, **p < 0.01, ***p < 0.001, ****p < 0.0001. **C** Representative Western blot of Cleaved PARP, Cleaved caspase-3, Bax and Bcl2 in ATDC5 chondrocytes pretreated with Lv-circFOXO3 with or without IL-1β (10 ng/mL) in the presence or absence of CQ (50 μM) or 3-MA (5 mM) for 24 h. **D** quantitative analysis of the protein levels in Fig. 7D. (*n* = 3) *p < 0.05, **p < 0.01, ***p < 0.001, ****p < 0.0001. **E** Representative Western blot of MMP13, ADAMTS5 and Collagen II in ATDC5 chondrocytes pretreated with Lv-circFOXO3 with or without IL-1β (10 ng/mL) in the presence or absence of CQ (50 μM) or 3-MA (5 mM) for 24 h. **F** quantitative analysis of the protein levels in Fig. 7F. (*n* = 3) *p < 0.05, **p < 0.01, ***p < 0.001, ****p < 0.0001. **G** PI3K/AKT, Phosphorylation of PI3K/AKT and Beclin1, LC3 in ATDC5 chondrocytes infected with Lv-circFOXO3 with or without IL-1β (10 ng/mL) for different time span. **H** Quantitative analysis of the protein levels in Figure 8B. (*n* = 3) *p < 0.05, **p < 0.01, ***p < 0.001, ****p < 0.0001.

We hypothesized that the regulation of autophagic activity by circFOXO3 may be involved in its regulation of chondrocyte apoptosis and ECM homeostasis. To investigate this hypothesis, we measured the levels of markers of apoptosis and ECM metabolism in ATDC5 chondrocytes in the absence and presence of 3-methyladenine (3-MA), an inhibitor of phosphatidylinositol 3-kinase (PI3K) that blocks autophagosome formation, or chloroquine (CQ), an inhibitor of autophagosome-lysosome fusion. The results showed that overexpression of circFOXO3 increased the protein level of Beclin1 and ratio of LC3II/I compared with that in the control, again suggesting a regulatory effect on autophagy (Fig. 6A, B). Furthermore, the rescue of IL-1β-induced autophagy inhibition by overexpression of circFOXO3 was reversed after the addition of an autophagy inhibitor (Fig. 6A, B). The ability of circFOXO3 to rescue the levels of apoptosis markers including BAX, Cleaved caspase-3, Cleaved PARP, and BCL-2 (Fig. 6C, D) as well as those of ECM metabolism markers including Collagen II, MMP13, and ADAMTS5 (Fig. 6E, F) was significantly reduced in the presence of the two autophagy inhibitors compared with that in control cells. These results suggest that the protective effect of circFOXO3 on chondrocytes is dependent on its autophagy-enhancing function.

### CircFOXO3 overexpression upregulates autophagy by inhibiting the PI3K/protein kinase B (AKT) pathway

To explore the molecular mechanisms underlying the effects of circFOXO3 on autophagy, we analyzed the level of the PI3K/AKT pathway following different exposure times (Fig. 6G). IL-1β (10 ng/ml) administration increased the ratio of p-PI3K/PI3K and p-AKT/AKT in a time-dependent manner between 30 min and 1 h compared with that in controls (Fig. 6G, H). However, overexpression of circFOXO3 counteracted the activation of p-PI3K and p-AKT in an inflammatory environment, suggesting that the regulation of autophagy by circFOXO3 may be achieved via partial inhibition of p-AKT activation. Furthermore, the LC3II/LC3I ratio and Beclin1 degradation were both increased in ATDC5 cells treated with 10 ng/ml IL-1β for 30 min to 1 h, while overexpression of circFOXO3 rescued the expression of these two autophagy markers compared with that in the control (Fig. 6G), suggesting that inhibition of the PI3K/AKT pathway is a major feature of circFOXO3-induced autophagy.

### Gene therapy with circFOXO3 preserves articular cartilage integrity in a mouse OA model

**Fig. 7:**
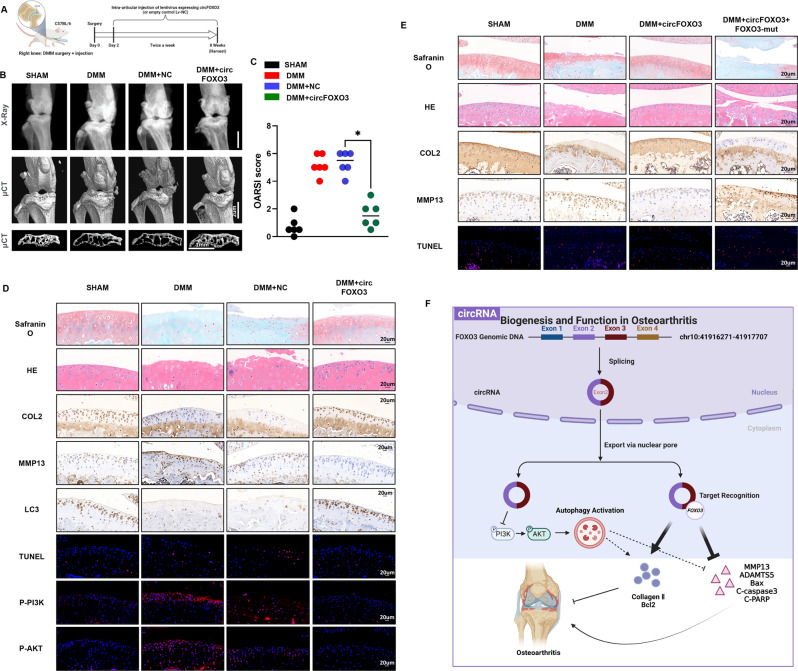
Gene therapy with circFOXO3 preserves articular cartilage integrity in a mouse OA model. **A** Schematic of the animal experiments (for each group, n = 6, C57BL/6). **B** X-ray and 3D reconstruction images of micro- CT scanning of the knees. Scale bars, 2 mm and 1 mm. **C** OARSI scoring was performed according to staining results. (*n* = 6) *p < 0.05. **D** Safranin-O/fast green staining, H&E staining, TUNEL staining, IHC staining and IF staining of cartilage from different groups. Scale bars, 20 μm. **E** Safranin-O/fast green staining, H&E staining, TUNEL staining and IHC staining of cartilage from different groups. Scale bars, 20 μm. **F** Graphic abstract of our study.

The mechanism identified above can potentially be applied in the treatment of OA. To evaluate whether circFOXO3 can serve as a target for OA therapy, a lentiviral vector encoding mouse circFOXO3 and the surgical destabilization of the DMM model of OA were used for animal studies in vivo (Fig. 7A). The lentivirus encoding circFOXO3 was injected into the mice two days after DMM surgery. Cartilage destruction was assessed eight weeks after DMM surgery by H&E staining, safranin-O staining, and OARSI grade. H&E and safranin-O staining showed that cartilage surfaces in DMM-induced OA mice were improved after injection of lentivirus encoding circFOXO3, but not mock lentivirus (Fig. 7D, top two). Quantitative analysis using OARSI scoring also revealed that Lv-circFOXO3 significantly lowered OARSI scores, whereas the DMM surgery and mock virus groups did not show improvements in the OARSI score (Fig. 7C).

Micro-CT is widely used to measure bone tissue because of its superiority over X-ray. Compared with the limitations of two-dimensional images, micro-CT performs 3D reconstruction of bone tissue, and more intuitively observes and measures data, such as bone density, bone morphology, and bone trabecular structure [34]. The 3D reconstruction of micro-CT and X-ray images demonstrated an increase in the number of osteophytes in OA mice compared with that in sham control mice, whereas circFOXO3 intra-articular injection decreased osteophytes (Fig. 7B). Additionally, the subchondral bone volume was also partially increased in the circFOXO3 injection group compared with that in the control (Fig. 7B). The injection of Lv-circFOXO3 alleviated the degenerative changes in the cartilage matrix, including the enhanced apoptotic (TUNEL) and catabolic responses and increased ECM composition in the OA mouse model compared with that in the sham, as indicated by immunohistochemistry and IF (Figs. 7D and S5A–C). Furthermore, using immunohistochemical analysis of LC3B, we further showed that the regulatory effect of circFOXO3 on OA progression is partly dependent on the autophagy mechanism in vivo (Fig. 7D, figure S5D). And in vivo experiments further proved that the regulation of circFOXO3 on autophagy is achieved through the PI3K/AKT signaling (Figs. 7D and S5E, F). These findings indicate that intra-articular circFOXO3 delivery may be a method for OA treatment, as it preserves cartilage integrity in the OA environment in vivo.

Finally, we further verified through in vivo experiments whether the regulatory effect of circFOXO3 on OA progression depends on its regulatory effect on FOXO3. H&E and safranin-O staining showed the substantially thickened cartilage layer on the articular surface after the injection of circFOXO3 overexpressing lentivirus; however, the knockdown of FOXO3 weakened this effect (Fig. 7E). Moreover, as indicated by immunohistochemistry (IHC), the injection of Lv-circFOXO3 decreased the expression of MMP13 and increased Collagen II levels in chondrocytes, while the knockdown of FOXO3 reduced the expression of Collagen II while increasing that of MMP13 (Figs. 7E and S6A, B). Furthermore, the cell apoptosis (TUNEL staining assay) showed consistent results as in vitro experiments (Figs. 7E and S6C). Together, our results indicated that circFOXO3 could alleviate OA in vivo by maintaining anabolism and inhibiting catabolism in the ECM of cartilage, and these effects were FOXO3-dependent.

## Discussion

OA is a degenerative joint disease characterized by progressive loss of articular cartilage. In OA, cartilage structure is reduced because of chondrocyte death and the remaining chondrocytes are activated by inflammatory cytokines, leading to the initiation of catabolic processes and abnormal differentiation, which degrade the ECM and further promote OA progression [35–37]. Elucidating the molecular mechanisms that control articular chondrocyte differentiation during articular cartilage development and maintenance may lead to the development of new therapeutic interventions. A more comprehensive understanding of the mechanisms of OA development and progression may aid the identification of these novel molecular targets.

CircRNA has received increasing attention owing to its important roles in disease occurrence, development, diagnosis, treatment, and prognosis, particularly in OA in recent years [38]. Two studies by Shen et al. [11, 39] revealed the protective roles of circPDE4B and circSERPINE2 on the cartilage in OA, respectively, suggesting that circRNA in chondrocytes may act on downstream targeted genes to protect articular cartilage. Wu et al. [40] reported that circPDE4D exerts its effect by acting as a sponge for miR-103a-3p and thereby regulates fibroblast growth factor 18 expression, to protect articular cartilage. Shen et al. [41] also demonstrated an important role for the circCDK14/miR-125a-5p/Smad2 axis in OA progression and provided a potential molecular therapeutic strategy for OA treatment. Together, these studies have demonstrated important protective effects of circRNAs in articular cartilage.

In the present study, we report that circFOXO3, which originates from exon 2 of FOXO3 and forms a ring structure by connecting the 3′ and 5′ splice sites, is highly expressed in normal articular cartilage and nearly absent in a surgically induced OA mouse model. Following RNase R treatment, the stability and integrity of circFOXO3 remained high and circFOXO3 was specifically expressed in cDNA and not gDNA after reverse transcription, which is consistent with circRNA characteristics [42–44]. In vitro, our results revealed that the expression of circFOXO3 was downregulated in cells treated with IL-1β compared with that in controls. Moreover, the staining results showed that circFOXO3 was negatively correlated with cartilage degeneration, suggesting that circFOXO3 may be involved in OA development. Further experiments indicated that circFOXO3 plays an important role in OA progression and may serve as a new target for its treatment.

Our findings demonstrated that circFOXO3 knockdown promotes increased expression of BAX, Cleaved PARP, and Cleaved caspase-3, and reduces the expression of BCL-2, suggesting that circFOXO3 inhibits apoptosis in chondrocytes. Conversely, overexpression of circFOXO3 attenuated IL-1β-induced apoptosis, suggesting that circFOXO3 partially inhibited apoptosis caused by inflammatory progression in the inflammatory environment of OA. Besides apoptosis, metabolic disturbance of the ECM is another important feature of OA [45–47]. Shen et al. [11] showed that circSERPINE2 upregulates the expression of collagen II, aggrecan, and other collagens in chondrocytes by interacting with downstream miR-1271, and inhibits the upregulation of MMPs such as MMP13. A study by Yang et al. [10] also showed that circRSU1 rescues an ECM metabolism disorder caused by oxidative stress. For the first time, we report that circFOXO3 may regulate ECM metabolism in OA. This study shows that circFOXO3 knockdown increases the levels of ADAMTS5 and MMP13 and reduces the levels of type II collagen, whereas circFOXO3 overexpression induces the opposite effect. Through subsequent experiments, we determined that the mechanism by which circFOXO3 regulates ECM metabolism is related to the autophagy and PI3K/AKT signaling cascade, and depends on its parental gene FOXO3.

There are four FOXO proteins in humans, namely FOXO1, FOXO3, FOXO4, and FOXO6, which all belong to the large family of forkhead box (Fox) transcription factors and share a highly conserved 100 amino acid DNA binding domain called the forkhead domain [48, 49]. FOXO protein activity plays a key role in cellular homeostasis and is tightly regulated. The most important regulation is the PI3K/AKT signaling pathway, which provides a critical inhibitory input signal for FOXO activity [50, 51]. Among the family members, FOXO1 and FOXO3 play major roles in cartilage homeostasis [52, 53]. FOXO3 is not only essential for bone development, but also protects against cartilage damage caused by aging [52] and further protects against OA by maintaining homeostasis of the meniscus during growth and development [53]. Here, we found that FOXO3 plays an important role in OA pathogenesis by regulating apoptosis in ATDC5 chondrocytes, further enriching its regulatory role in OA homeostasis. We found that the regulation of circFOXO3 in OA is achieved through FOXO3. Therefore, considering that circFOXO3 was found to function through FOXO3, emphasis on the important role of the circFOXO3-FOXO3 axis in ECM metabolism and apoptosis in OA will help to further explore the circFOXO3 mechanism.

Autophagy helps cells digest and decompose their own components to maintain a dynamic and stable intracellular environment. When cells are under stress, autophagy helps them adapt to new environmental conditions to ensure the availability of compounds necessary to synthesize macromolecules for survival, and moderate autophagy can rescue apoptosis and cell death [6]. The occurrence and development of several diseases, including tumors, kidney diseases, liver diseases, and cerebrovascular diseases, are closely related to autophagy, and this process is likely to become a new therapeutic entry point in disease progression [12, 54–57]. In this study, we demonstrate that circFOXO3 in articular cartilage regulates the apoptosis level of cells in vivo and in vitro and the metabolic homeostasis of ECM through autophagy. Moreover, circFOXO3 is likely to activate autophagy via inhibition of the PI3K/AKT pathway. Suppression of the PI3K/AKT pathway may lead to cell survival or death via autophagy or apoptosis, respectively [6, 13, 58]. These results indicate that circFOXO3 may be linked to autophagy in the pathological process of OA, which is consistent with the related research conclusions about circFOXO3 and autophagy in other diseases [12].

Our study also has certain limitations. As mentioned in other studies, our conclusions would be more convincing if we constructed transgenic circFOXO3 knockout mice rather than simply injecting overexpressing lentivirus into the joint cavity of mice [10]. However, our method may also be improved by similar methods of drug delivery, including wrapping exosomes or hydrogels around circRNA molecules—a common method for stable delivery of therapeutic contents [59, 60]. Second, we used the ATDC5 cell line instead of primary chondrocytes in our cellular experiments. However, the ATDC5 cell line is often used as a cellular model to assess chondrogenesis in vitro [29]. After the addition of ITS to the medium, the cells can form cartilage nodule-like cell aggregates that can produce specific proteoglycans and type II collagen, typical molecular markers of chondrocytes. ATDC5, therefore, provides a good model with which to understand chondrogenic differentiation. In addition, the ATDC5 chondrocytes has been widely used as a cell research tool in many osteoarthritis studies in recent years [30, 47, 61, 62], which has good chondrocyte characteristics while killing as few animals as possible. Furthermore, although our study demonstrated that the regulation of autophagy by circFOXO3 may play a key role in the occurrence and progression of OA, our results do not rule out the possibility that other molecules that interact with circFOXO3 may be involved in autophagy activation. Yang et al. [26] showed that circFOXO3 interacts with mammalian target of rapamycin (mTOR) and E2F1 to suppress mTOR complex 1 activity, which promotes autophagy. This mechanism may also be involved in the regulation of circFOXO3 in OA. To understand the mechanism by which circFOXO3 improves OA conditions by activating autophagy, future studies should focus on exploring other molecules related to circFOXO3 and autophagy functions.

In conclusion, our research unveiled a new signaling pathway, circFOXO3/FOXO3, that may protect against OA by maintaining the ECM of chondrocytes and regulating chondrocyte apoptosis and proliferation (Fig. 7F). Consequently, overexpression of circFOXO3 may be a promising therapeutic approach for treating OA.

## Supplementary information

Supplementary figure legends

Original western blots

checklist

Figure S1

Figure S2

Figure S3

Figure S4

Figure S5

Figure S6

## Data Availability

All data generated or analyzed during this study are included in this published article.
