# Supplementary material for: CircFOXO3 protects against osteoarthritis by targeting its parental gene FOXO3 and activating PI3K/AKT-mediated autophagy
Source: Cell Death Dis. 2022 Nov 7;13(11):932. doi: 10.1038/s41419-022-05390-8 (PMC9640610; doi:10.1038/s41419-022-05390-8)
Supplement: Supplementary file 1 — Supplementary figure legends [file 41419_2022_5390_MOESM1_ESM.docx]

Supporting figure legends

**CircFOXO3 protects against osteoarthritis by targeting its parental gene FOXO3 and activating PI3K/AKT-mediated autophagy**

The supporting information includes:

• Supplementary figure legends

**Figure S1. The downstream molecule FOXO3 regulates apoptosis and ECM metabolism in ATDC5 chondrocytes.** (A) The overexpression and knock down efficiency of FOXO3 in ATDC5s detected by qRT-PCR. (n=3) **p<0.01, ***p<0.001, ****p<0.0001. (B, C) Western blot analysis of the overexpression and knock down efficiency of FOXO3. The optical density analysis was performed from the results of three independent experiments of western blot samples. **p<0.01, ***p<0.001, ****p<0.0001. (D) Cell viability determined by CCK-8 assay. (n=3) **p<0.01, ***p<0.001, ****p<0.0001. (E) Alcian blue staining of siRNA-treated ATDC5 chondrocytes. (F, G) Western blot analysis of Cleaved PARP, Cleaved caspase3, Bax and Bcl2 when FOXO3 was downregulated or upregulated in ATDC5 chondrocytes. The optical density analysis was performed from the results of three independent experiments of western blot samples. *p<0.05, **p<0.01, ***p<0.001, ****p<0.0001. (H, I) Western blot analysis of MMP13, ADAMTS5 and Collagen II when FOXO3 was downregulated or upregulated in ATDC5 chondrocytes. The optical density analysis was performed from the results of three independent experiments of western blot samples. *p<0.05, **p<0.01, ***p<0.001. (J) Alcian blue staining of lenti-virus-treated ATDC5 chondrocytes. (K, L) ATDC5s proliferation activity was detected by TUNEL staining when FOXO3 was overexpressed. Representative photomicrographs and quantitative data showing the percentage of TUNEL-positive cells are shown. (n=3) Scale bar, 200 µm. **p<0.01, ***p<0.001. (M, N) Representative photomicrographs and fluorescence intensity of IF of MMP13, and Collagen II in ATDC5s after treating with IL-1β (10 ng/ml) for 24 hours and the saving effects of overexpressed FOXO3 on IL-1β. (n=3) Scale bar, 25 µm. **p<0.01, ***p<0.001, ****p<0.0001.

**Figure S2.** **Effects of overexpression of FOXO3 on ECM metabolism.**

**Figure S3.** **Effects of knockdown of FOXO3 on apoptosis and ECM metabolism.** (A, B) ATDC5s proliferation activity was detected by TUNEL staining when FOXO3 was knocked down. Representative photomicrographs and quantitative data showing the percentage of TUNEL-positive cells are shown. (n=3) Scale bar, 100 µm. (C, D) Representative photomicrographs and fluorescence intensity of IF of Collagen II in ATDC5s after treating with IL-1β (10 ng/ml) for 24 hours and the worsening effects of knock down FOXO3 on IL-1β. (n=3) Scale bar, 25 µm. *p<0.05, **p<0.01.

**Figure S4.** **The effect of circFOXO3 on apoptosis and ECM metabolism is achieved by acting on FOXO3.** (A) Quantitative data showing the percentage of TUNEL-positive cells are shown. (n=3) (B, C) The optical density analysis was performed from the results of three independent experiments of western blot samples. *p<0.05, **p<0.01, ***p<0.001, ****p<0.0001.

**Figure S5.** **Quantitative analysis of TUNEL staining and IHC staining.** (A) Quantitative data showing the percentage of TUNEL-positive cells are shown. (n=6) (B- D) Histological analysis of mouse cartilage tissue in each group. LC3, MMP13 and Collagen II expression was examined by immunohistochemistry. (n=6) (E- F) IF analysis of mouse cartilage tissue in each group. P-PI3K and P-AKT expression was examined by immunofluorescence. (n=6) ***p<0.001, ****p<0.0001.

**Figure S6.** **Quantitative analysis of TUNEL staining and IHC staining.** (A- B) Histological analysis of mouse cartilage tissue in each group. MMP13 and Collagen II expression was examined by immunohistochemistry. (n=6) (C) Quantitative data showing the percentage of TUNEL-positive cells are shown. (n=6) *p<0.05, ****p<0.0001.
