# Supplementary material for: CircFOXO3 protects against osteoarthritis by targeting its parental gene FOXO3 and activating PI3K/AKT-mediated autophagy
Source: Cell Death Dis. 2022 Nov 7;13(11):932. doi: 10.1038/s41419-022-05390-8 (PMC9640610; doi:10.1038/s41419-022-05390-8)
Supplement: Supplementary file 2 — Original western blots [file 41419_2022_5390_MOESM2_ESM.docx]

Supplemental Material

**CircFOXO3 protects against osteoarthritis by targeting its parental gene FOXO3 and activating PI3K/AKT-mediated autophagy**

The supporting information includes:

• Original western blots-colored images


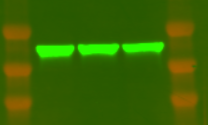


**β-actin**


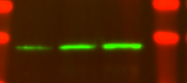


**BAX**


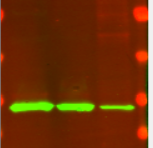


**BCL-2**


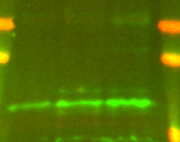


**Cleaved caspase3**


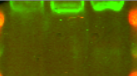


**Cleaved PARP**

**Figure 2D. The effect of circFOXO3 knockdown on chondrocyte apoptosis**


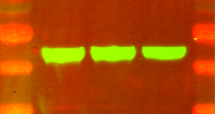


**β-actin**


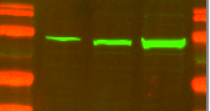


**ADAMTS5**


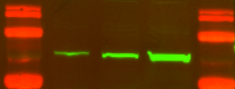


**MMP13**


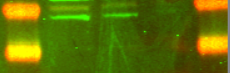


**Collagen Ⅱ**

**Figure 2F. The effect of circFOXO3 knockdown on ECM metabolism.**


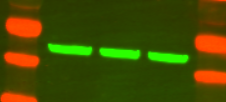


**β-actin**


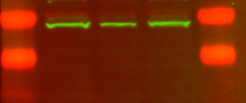


**BCL-2**


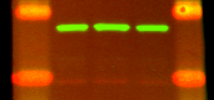


**BAX**


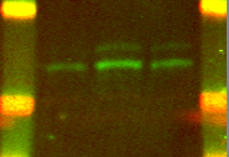


**Cleaved caspase3**


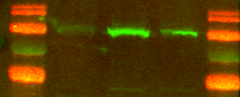


**Cleaved PARP**

**Figure 3D. The effect of circFOXO3 overexpression on chondrocyte apoptosis.**


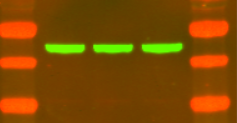


**β-actin**


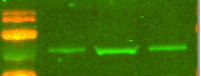


**MMP13**


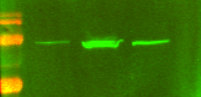


**ADAMTS5**


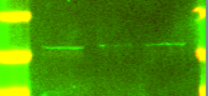


**Collagen Ⅱ**

**Figure 3F. The effect of circFOXO3 overexpression on ECM metabolism.**


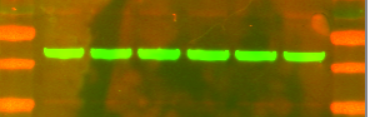


**β-actin**


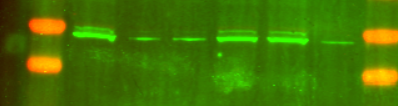


**BCL-2**


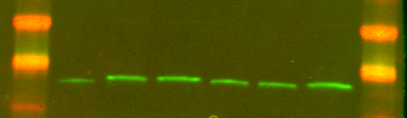


**BAX**


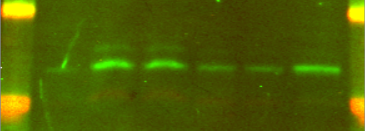


**Cleaved caspase3**


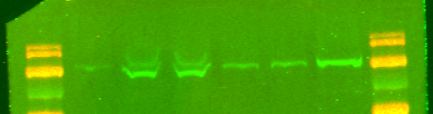


**Cleaved PARP**

**Figure 4D. Regulation of apoptosis by circFOXO3 is FOXO3-dependent.**


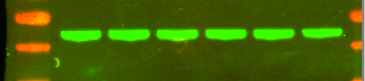


**β-actin**


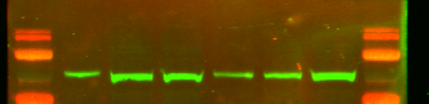


**MMP13**


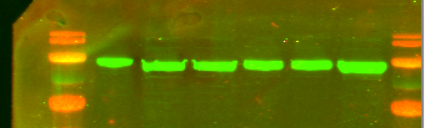


**ADAMTS5**


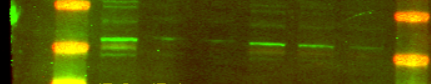


**Collagen Ⅱ**

**Figure 4D. Regulation of ECM metabolism by circFOXO3 is FOXO3-dependent.**


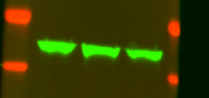


**β-actin**


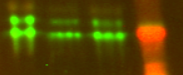


**LC3**


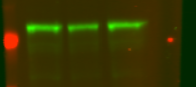


**BECLIN1**

**Figure 5C. CircFOXO3 enhances autophagy in IL-1β-treated ATDC5 chondrocytes.**


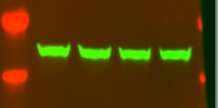


**β-actin**


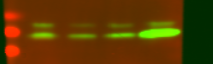


**LC3**


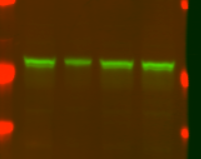


**BECIN1**


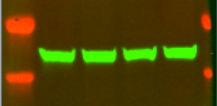


**β-actin**


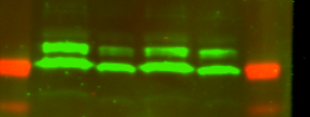


**LC3**


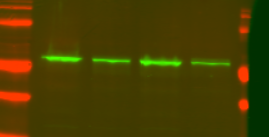


**BELIN1**

**Figure 6A. CircFOXO3-enhanced autophagy is reversed by autophagy inhibitors.**


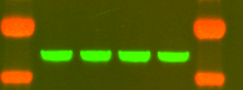


**β-actin**


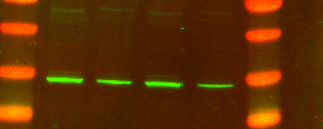


**BCL-2**


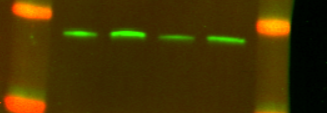


**BAX**


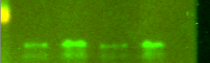


**Cleaved caspase3**


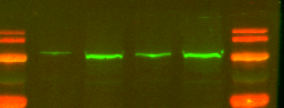


**Cleaved PARP**


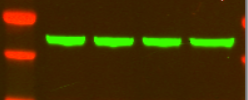


**β-actin**


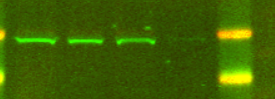


**BCL2**


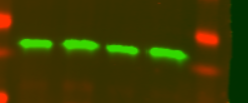


**BAX**


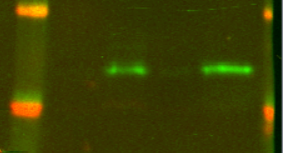


**Cleaved caspase3**


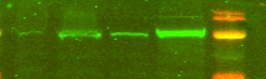


**Cleaved PARP**

**Figure 6C. CircFOXO3 autophagy sensitively regulates the IL-1β-induced ATDC5 chondrocyte apoptosis.**


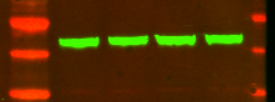


**β-actin**


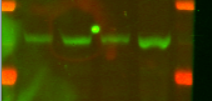


**MMP13**


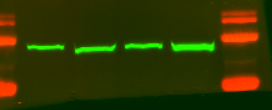


**ADAMTS5**


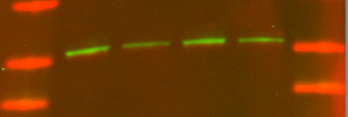


**Collagen Ⅱ**


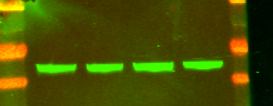


**β-actin**


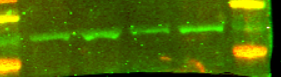


**MMP13**


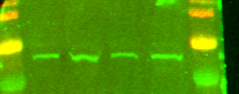


**ADAMTS5**


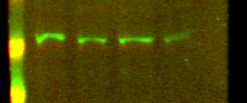


**Collagen Ⅱ**

**Figure 6E. CircFOXO3 autophagy sensitively regulates the IL-1β-induced ATDC5 chondrocyte ECM dysregulated.**


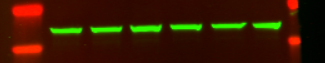


**β-actin**


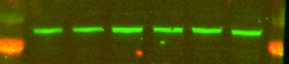


**AKT**


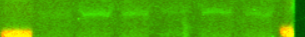


**p-AKT**


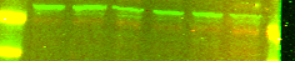


**PI3K**


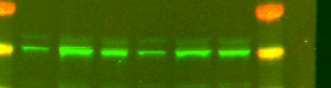


**p-PI3K**


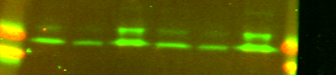


**LC3**


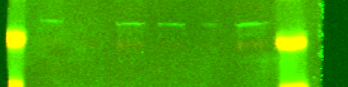


**BECLIN1**

**Figure 6G. CircFOXO3 overexpression upregulates autophagy by inhibiting the PI3K/protein kinase B (AKT) pathway.**


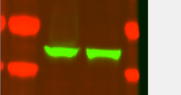


**actin**


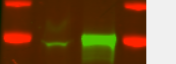


**FOXO3**

**Supplementary figure 1 B. Validation of the success of FOXO3 overexpression lentivirus by WB.**


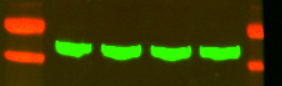


**actin**


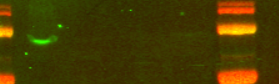


**FOXO3**

**Supplementary figure 1 B. Validation of the success of FOXO3 knockdown by WB.**


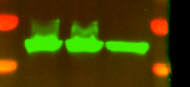


**actin**


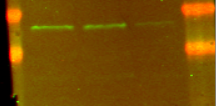


**BCL-2**


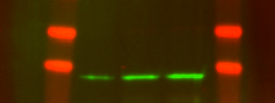


**BAX**


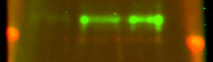


**Cleaved caspase3**


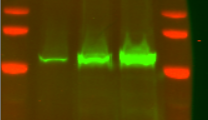


**Cleaved PARP**

**Supplementary figure 1 F. The effect of FOXO3 knockdown on chondrocyte apoptosis.**


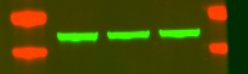


**actin**


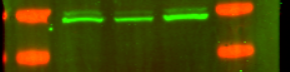


**BCL2**


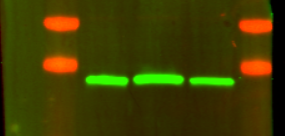


**BAX**


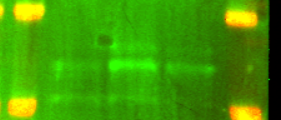


**Cleaved caspase3**


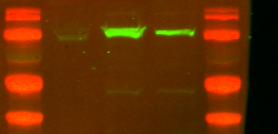


**Cleaved PARP**

**Supplementary figure 1 F. The effect of FOXO3 overexpression on chondrocyte apoptosis.**


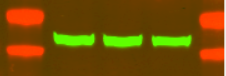


**actin**


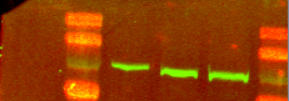


**MMP13**


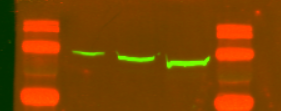


**ADAMTS5**


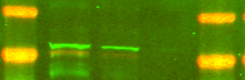


**Collagen Ⅱ**

**Supplementary figure 1 H. The effect of FOXO3 knockdown on ECM metabolism.**


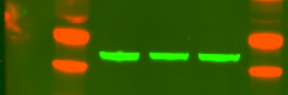


**actin**


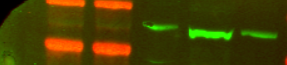


**MMP13**


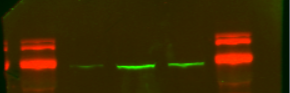


**ADAMTS5**


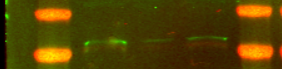


**Collagen Ⅱ**

**Supplementary figure 1 H. The effect of FOXO3 overexpression on ECM metabolism.**


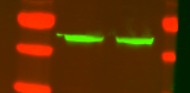


**actin**


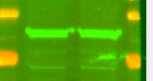


**MMP13**


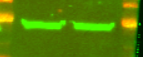


**ADAMTS5**


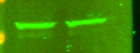


**Collagen Ⅱ**

**Supplementary figure 2.**
