# Supplementary figures and images for: CircFOXO3 protects against osteoarthritis by targeting its parental gene FOXO3 and activating PI3K/AKT-mediated autophagy
Source: Cell Death Dis. 2022 Nov 7;13(11):932. doi: 10.1038/s41419-022-05390-8 (PMC9640610; doi:10.1038/s41419-022-05390-8)

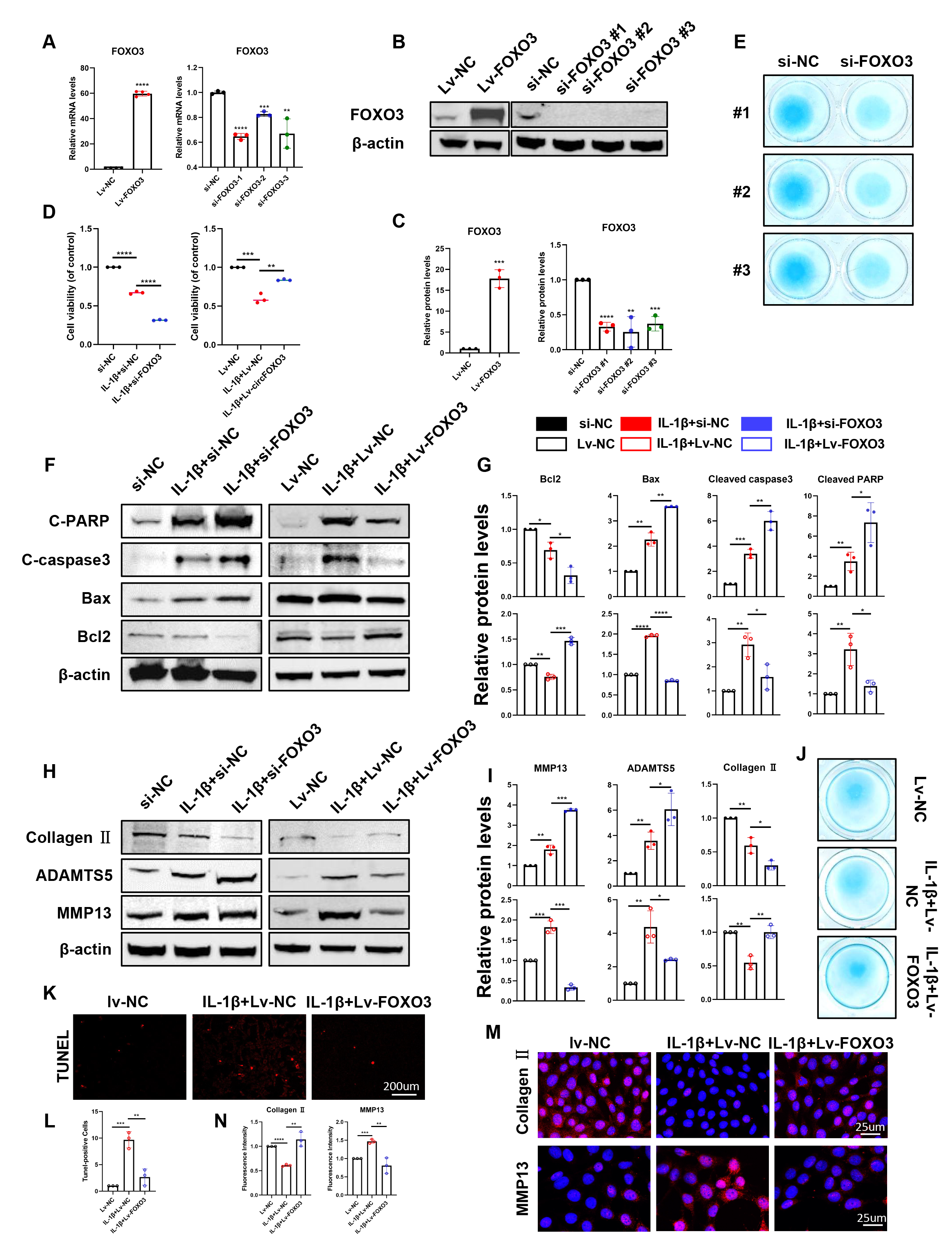

Supplement: Supplementary file 4 — Figure S1 [file 41419_2022_5390_MOESM4_ESM.tif]

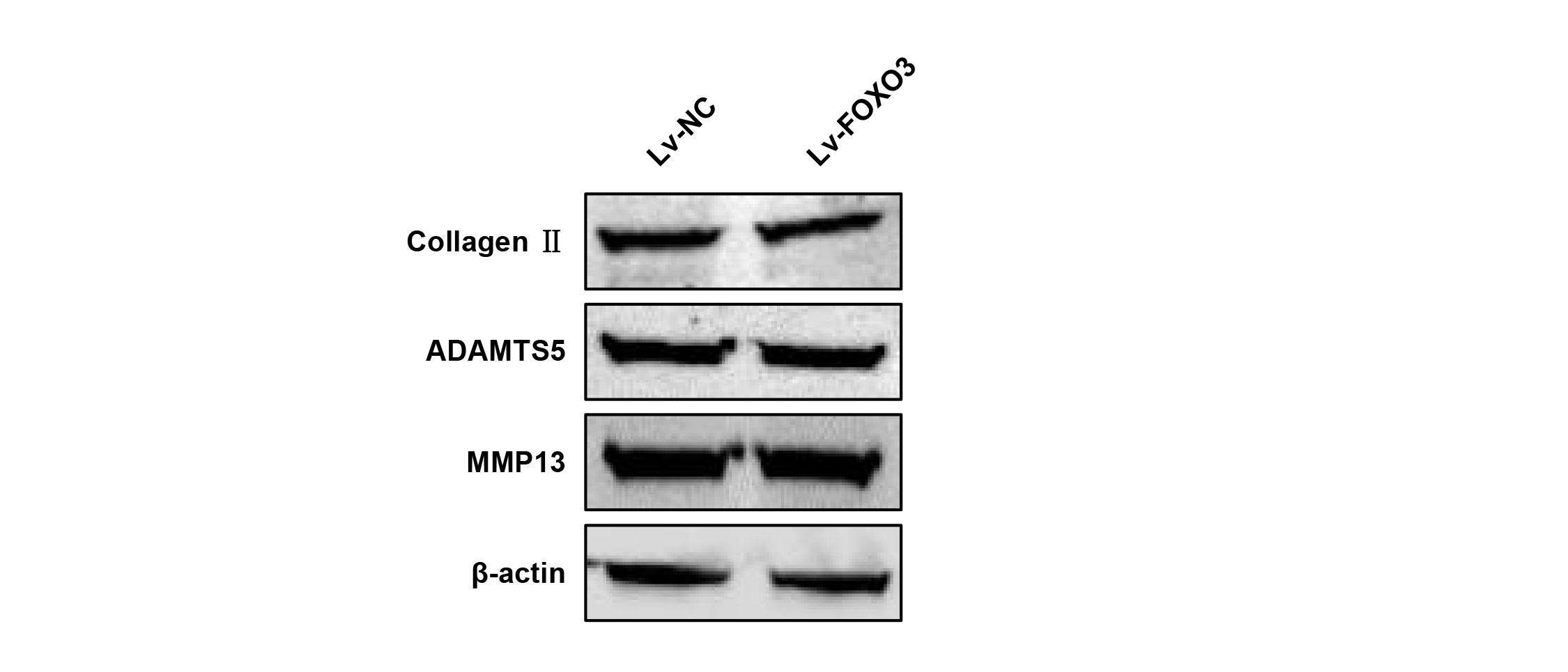

Supplement: Supplementary file 5 — Figure S2 [file 41419_2022_5390_MOESM5_ESM.tif]

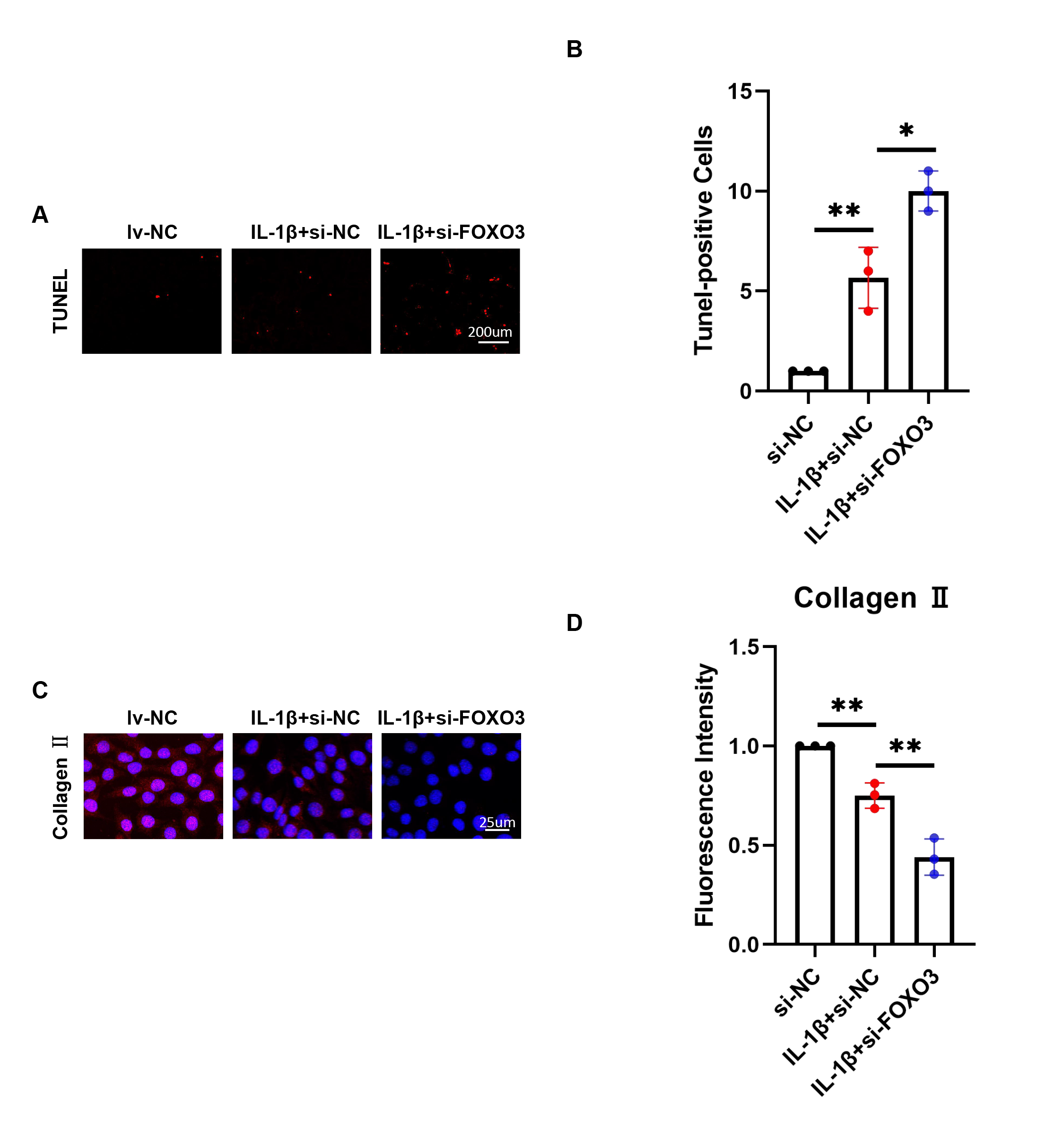

Supplement: Supplementary file 6 — Figure S3 [file 41419_2022_5390_MOESM6_ESM.tif]

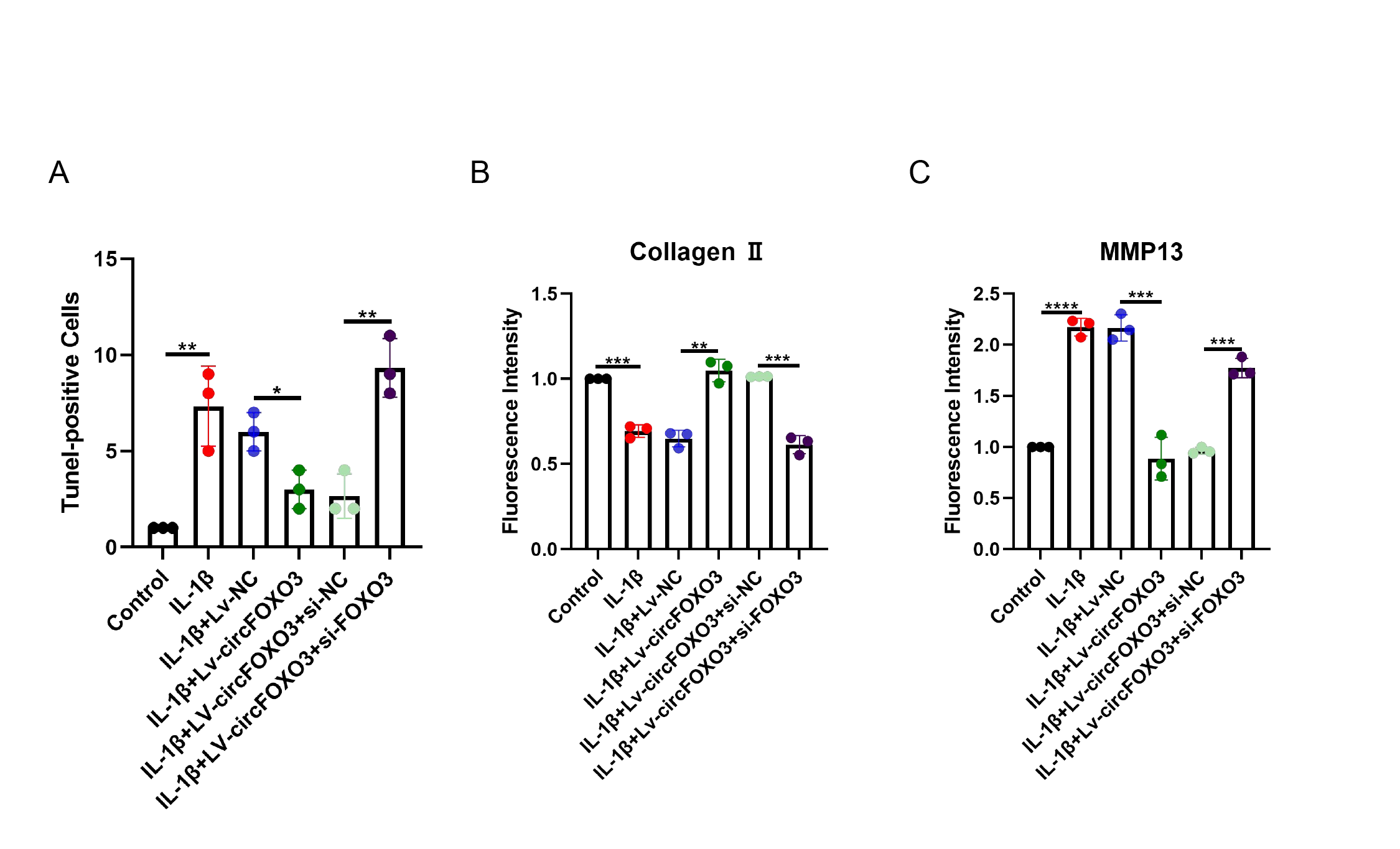

Supplement: Supplementary file 7 — Figure S4 [file 41419_2022_5390_MOESM7_ESM.tif]

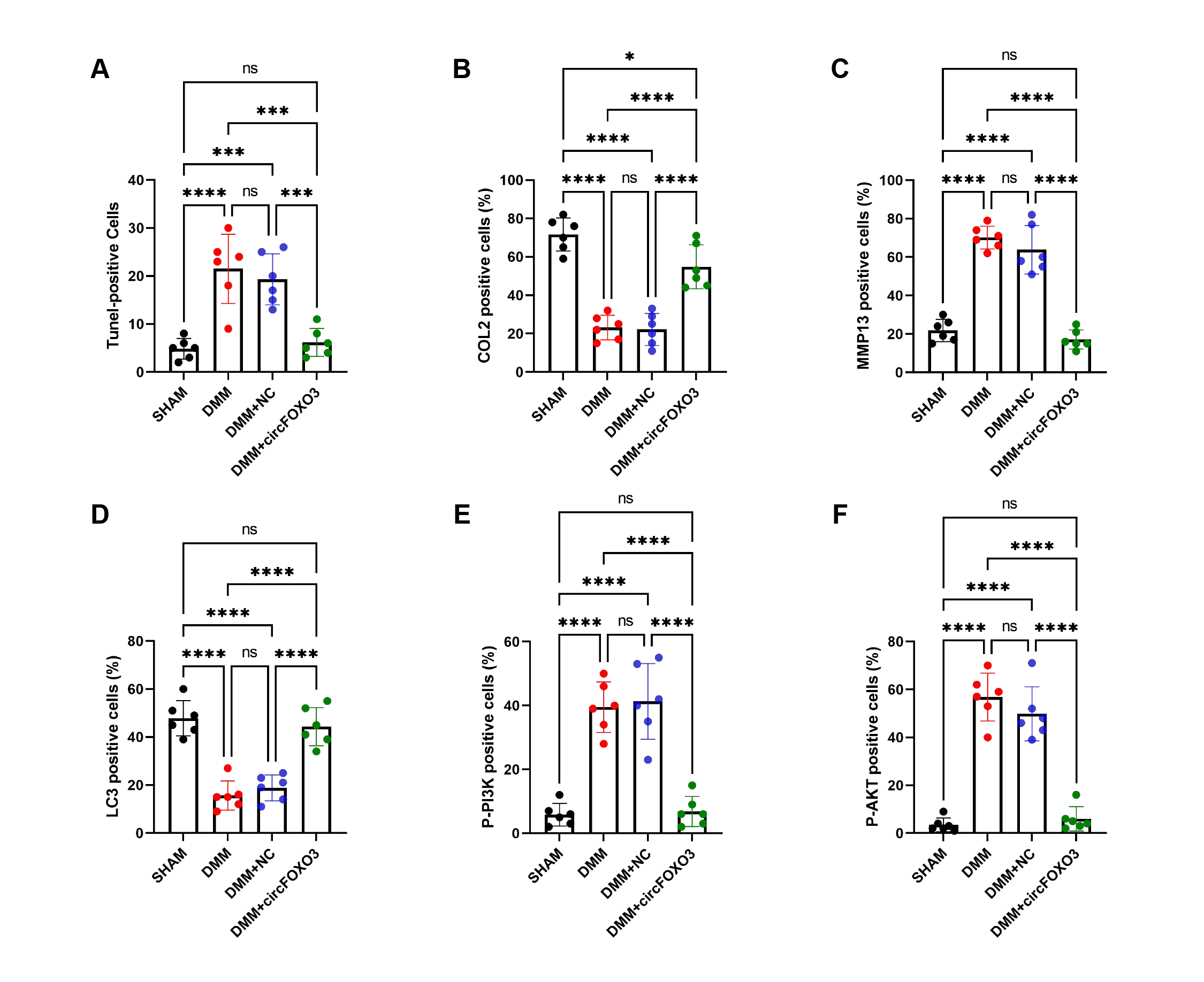

Supplement: Supplementary file 8 — Figure S5 [file 41419_2022_5390_MOESM8_ESM.tif]

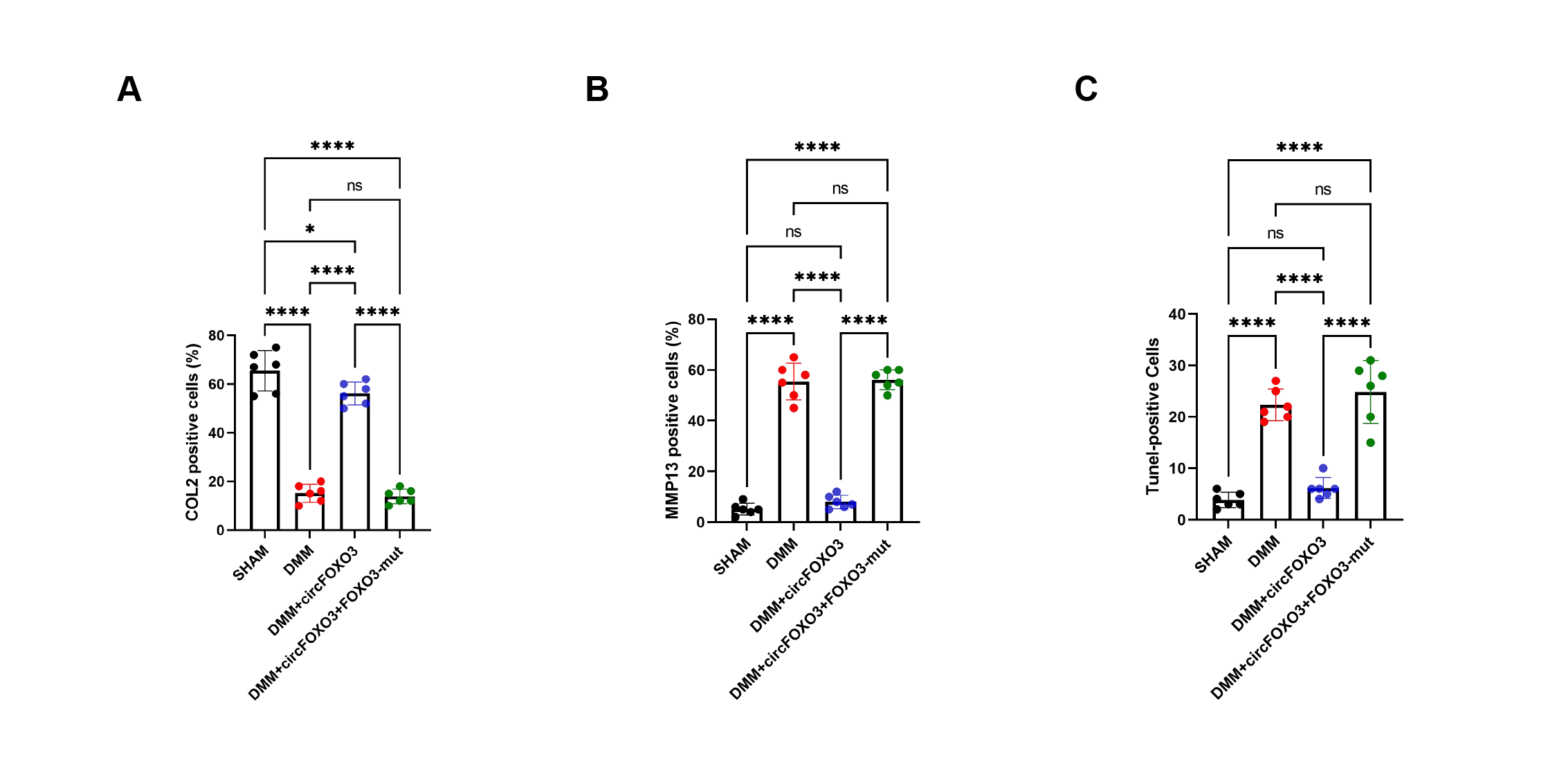

Supplement: Supplementary file 9 — Figure S6 [file 41419_2022_5390_MOESM9_ESM.tif]
